# Liver-targeted polymeric prodrugs delivered subcutaneously improve tafenoquine therapeutic window for malaria radical cure

**DOI:** 10.1126/sciadv.adk4492

**Published:** 2024-04-19

**Authors:** Ayumi E. Pottenger, Debashish Roy, Selvi Srinivasan, Thomas E. J. Chavas, Vladmir Vlaskin, Duy-Khiet Ho, Vincent C. Livingston, Mahdi Maktabi, Hsiuling Lin, Jing Zhang, Brandon Pybus, Karl Kudyba, Alison Roth, Peter Senter, George Tyson, Hans E. Huber, David Wesche, Rosemary Rochford, Paul A. Burke, Patrick S. Stayton

**Affiliations:** ^1^ Department of Bioengineering, University of Washington, Seattle, WA 98195, USA.; ^2^ Department of Immunology and Microbiology, University of Colorado Anschutz School of Medicine, Aurora, CO 80045, USA.; ^3^ Department of Drug Discovery, Experimental Therapeutics Branch, Walter Reed Army Institute of Research, Silver Spring, MD 20910, USA.; ^4^ Seagen, Bothell, WA 98021, USA.; ^5^ George Tyson Consulting, Los Altos Hills, CA 94022, USA.; ^6^ Department of Chemical and Biomolecular Engineering, University of California, Berkeley, Berkeley, CA 94720, USA.; ^7^ BioTD Strategies LLC, 213 Abbey Ln., Lansdale, PA 19446, USA.; ^8^ Certara, Princeton, NJ 08540, USA.; ^9^ Burke Bioventures LLC, 1 Broadway 14th Floor, Cambridge, MA 02142, USA.

## Abstract

Approximately 3.3 billion people live with the threat of *Plasmodium vivax* malaria. Infection can result in liver-localized hypnozoites, which when reactivated cause relapsing malaria. This work demonstrates that an enzyme-cleavable polymeric prodrug of tafenoquine addresses key requirements for a mass administration, eradication campaign: excellent subcutaneous bioavailability, complete parasite control after a single dose, improved therapeutic window compared to the parent oral drug, and low cost of goods sold (COGS) at less than $1.50 per dose. Liver targeting and subcutaneous dosing resulted in improved liver:plasma exposure profiles, with increased efficacy and reduced glucose 6-phosphate dehydrogenase–dependent hemotoxicity in validated preclinical models. A COGS and manufacturability analysis demonstrated global scalability, affordability, and the ability to redesign this fully synthetic polymeric prodrug specifically to increase global equity and access. Together, this polymer prodrug platform is a candidate for evaluation in human patients and shows potential for *P. vivax* eradication campaigns.

## Teaser

A liver-targeted, enzyme-cleavable antimalarial polymeric prodrug improved efficacy and reduced hemotoxicity in mouse models.

## INTRODUCTION

There were an estimated 247 million cases of malaria and 619,000 malaria-related deaths in 2021, and African leaders have recently warned the UN General Assembly that the worst malaria crisis in two decades could be on the horizon (*1*, *2*). While *Plasmodium falciparum* is responsible for the highest number of malarial deaths, *Plasmodium vivax* is the most widespread of the *Plasmodium* species, with roughly 3.3 billion of the global population living within the limits of *P. vivax* transmission (*3*). *P. vivax* is the dominant species in areas including the Americas, the Greater Mekong Subregion, India, most countries within the World Health Organization (WHO) South-East Asia Region, and most countries within the WHO Western Pacific Region (*2*). Climate change is widely predicted to worsen the *P. vivax* threat across the eastern Mediterranean and broad Asian geographic regions (*4*, *5*). In some countries where *P. falciparum* and *P. vivax* are co-endemic such as Myanmar, Vietnam, Lao People’s Democratic Republic, and Cambodia, *P. vivax* has become the dominant species between 2010 and 2021 (*2*, *3*). *P. vivax* infections can result in dormant liver-stage hypnozoites, which are not eradicated with standard malaria treatments (*6*). These infections carry an especially heavy individual and public health burden among pregnant women and children (*7*–*12*). Malarial relapse from dormant hypnozoites also contributes to the continued transmission of *P. vivax* (*13*).

New therapeutic approaches that meet the needs for mass administration and eradication campaigns are thus badly needed. The 8-aminoquinoline (8-AQ) drugs primaquine (PQ) and tafenoquine (TQ) are the only approved therapies for radical cure, i.e., elimination of liver hypnozoites, of *P. vivax* (*14*). TQ was approved by the US Food and Drug Administration (FDA) in 2018 as a single-dose oral therapy, which was an exciting improvement compared to the standard 14-day PQ standard of care (*15*–*22*). TQ thus has the potential for mass administration and global eradication campaigns. However, the primary issue with TQ-based eradication campaigns remains the contraindication of 8-AQs for those with glucose-6-phosphate dehydrogenase (G6PD) deficiency or unknown G6PD status. G6PD deficiency is one of the most common human enzymopathies—ironically due to its historical evolutionary advantages against malaria disease (*23*, *24*)—and affects 400 million people worldwide, with G6PD deficiency alleles occurring with a frequency as high as 16.8% of the population in some malaria-endemic regions (*23*, *25*, *26*). The 8-AQs and their metabolites cause oxidative stress to red blood cells (RBCs), resulting in severe anemia, renal failure, and even death in patients with severe G6PD deficiency (*27*). The FDA requires prior testing and absence of G6PD deficiency before prescribing TQ. This testing is not easily accessible in resource-poor regions where malaria is most common (*28*), making TQ usage problematic in designing malaria eradication campaigns. In addition, the contraindication for G6PD deficiency removes a significant fraction of potential patients, leaving a latent pool of *P. vivax* carriers that would make mass eradication problematic. The prodrug approach outlined here is designed to address both TQ usage limitations.

Despite decades of 8-AQ research, the exact mechanisms and active metabolites responsible for antiparasitic activity and hemolytic anemia remain elusive (*29*). The involvement of redox-active metabolites in both efficacy and toxicity suggested that the two activities may be mechanistically linked and inseparable (*30*). Earlier preclinical efforts to reduce the toxicity of 8-AQ via formulation or delivery approaches have not demonstrated an increase in the therapeutic window (*31*–*35*). However, a recent study in healthy Thai women suggested that even a relatively small improvement in the therapeutic index may be clinically meaningful and allow use of TQ in G6PD-deficient patients. Female volunteers heterozygous for the Mahidol*^487A^* G6PD–deficient variant were compared with G6PD-normal women. The study showed dose-dependent hemolytic toxicity in the G6PD-deficient patients, as defined by decreases in hemoglobin (Hgb) and hematocrit (*36*). Whereas the approved dose of 300-mg TQ was dose-limiting in the G6PD-deficient cohort, a 100-mg dose did not produce hemotoxicity. A TQ dose of 300 mg may be insufficient for radical cure based on regional differences in response (*37*, *38*), and this study begins to identify the magnitude of therapeutic index improvement needed to avoid the G6PD deficiency contraindication and testing requirements.

**Fig. 1.**
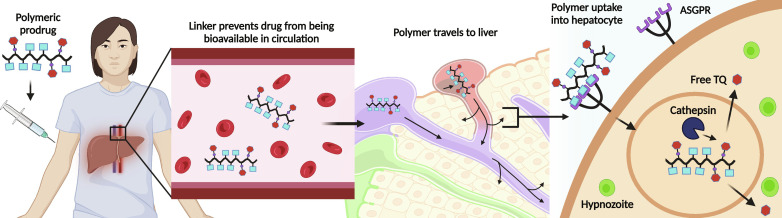
Conceptual overview of liver-targeted polymeric prodrug delivery mechanism. The polymer prodrug with TQ is singly dosed SC and traffics in circulation to the liver. The polymeric prodrug achieves targeting and receptor-mediated endocytosis in hepatocytes via its GalNAc glycan ligand that is also incorporated into the polymer as a functionalized monomer. The blood-stabilized valine-citrulline linker is enzymatically cleaved by cathepsin enzymes in the endosomal-lysosomal compartments, and the TQ payload is released into the cytoplasm to kill dormant hypnozoites. Created with Biorender.com.

Here, we describe a liver-targeted, polymeric prodrug approach (*39*) for increasing the TQ therapeutic index (Fig. 1). The subcutaneously (SC) administered prodrugs are designed with blood-stable linkers to lower TQ blood *C*_*max*_ (maximum TQ concentration) and thus reduce hemotoxicity, to maximize intra-hepatocyte drug exposure of hypnozoites to achieve radical cure with a single dose, and to alter the oral gut and first-pass liver metabolism of TQ that is connected with hemotoxicity in the blood RBC compartment (*16*, *40*, *41*). More specifically, the latter hypothesis was to reduce the circulating TQ metabolites produced after oral drug administration and rapid gut/liver TQ metabolism. Liver metabolism is, however, also required for anti-hypnozoite activity, and thus, it was a question whether the alternative parenteral SC administration route, using a single-dose requirement for clinical mass administration, could indeed achieve liver TQ levels necessary for preliminary efficacy validation. The targeting of hepatocytes and the intracellular hypnozoite reservoir was designed with the *N*-acetyl galactosamine (GalNAc) ligand against the asialoglycoprotein receptor (ASGPR) as validated in human-approved small interfering RNA (siRNA) products (*42*, *43*). This parenteral prodrug design also exploits the brentuximab vedotin antibody-drug conjugate (ADC) linker design to achieve high blood stability and intracellular drug release (*44*, *45*). Prior work has shown that these polymer prodrugs could achieve relevant liver drug exposures by intravenous (IV) injection routes, but this is not a viable global health administration method for mass administration requirements (*46*). The less stable valine-citrulline linker used previously has been shown to be cleavable in mouse blood with ADCs by extracellular carboxylesterases, leading to increases in blood drug exposures (*47*, *48*).

 These cathepsin-cleavable, SC administered polymeric prodrugs are shown to achieve high liver hepatocyte selectivity and high blood stability (i.e., low drug release in blood). While a new orally administered radical cure drug would be most desirable if it did not pose safety issues in G6PD-deficient humans, there are no current candidates in clinical trials. The portability of SC devices opens the possibility that a single-dose SC injectable drug could still meet the distributed clinical administration requirements for mass antimalarial eradication campaigns (*49*). Efficacy was improved relative to oral TQ against *Plasmodium berghei* sporozoite infections. They also significantly reduced hemotoxicity in a humanized mouse model of G6PD deficiency, using human G6PD-deficient blood donors. A major challenge in radical cure drug development is the lack of *P. vivax* anti-hypnozoite animal models. While there is a nonhuman primate (NHP) model of *Plasmodium cynomolgi* hypnozoites, this model is not suited for the comparative assessment of TQ prodrugs because of differences in TQ metabolism and pharmacokinetic/pharmacodynamic (PK/PD) correlations between NHP versus humans (*50*). In addition, use of NHP in drug development has been constrained by availability and supply (*51*). We thus have demonstrated the anti-hypnozoite activity of the optimized polymeric prodrug in an established ex vivo model of radical cure, developed as a drug screening platform, that incorporates primary NHP hepatocytes and *P. cynomolgi* hypnozoites (*52*). Last, we have performed a cost of goods sold (COGS) and manufacturability analysis for these polymeric prodrugs to examine whether they are feasible at the scale and COGS required for translatability.

## RESULTS

### Prodrug monomer without PABC spacer designed, synthesized, and used in RAFT polymerization to make an optimized polymeric prodrug

**Fig. 2.**
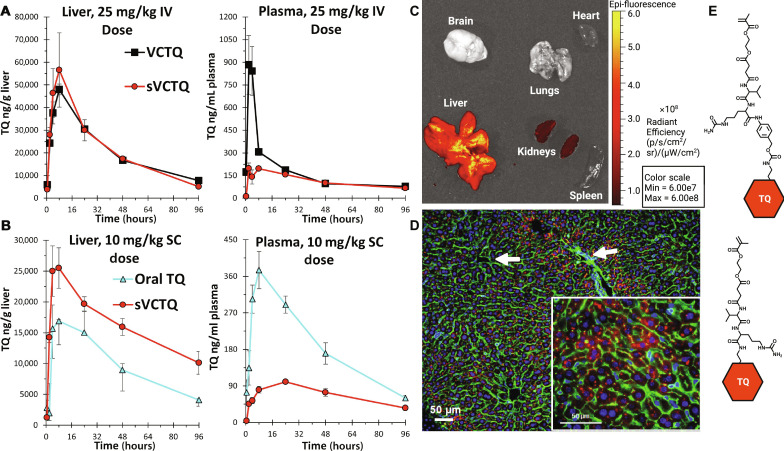
pSVCTQ results in optimal TQ release, polymer biodistribution specificity to liver, and subcellular hepatocyte localization. (**A**) Liver and plasma concentrations of TQ after 25 mg/kg TQ equivalent dose of pSVCTQ or pVCTQ via IV route of administration. pSVCTQ dose results in lower plasma *C*_max_ compared to pVCTQ dose. (**B**) Liver and plasma concentrations of TQ after 10 mg/kg TQ equivalent dose of pSVCTQ via SC route of administration or 10 mg/kg oral dose of free TQ. pSVCTQ dose results in greater liver exposure and reduced plasma exposure compared to oral TQ. (**C**) IVIS imaging of relevant tissues at 8 hours post-SC 25 mg/kg dose of fluorescent rhodamine-labeled pSVCTQ. (**D**) Representative image of liver tissues prepared for imaging. Liver was collected at 8 hours post-SC 25 mg/kg dose of fluorescent rhodamine-labeled pSVCTQ. Five-micrometer-thick sections were prepared using CryoStat at −20°C, and the sections were counterstained with DAPI to visualize nuclei (blue) and Alexa 488 Phalloidin to visualize cell outlines (green). The polymer is in red. White arrows point to sinusoidal lumen. (**E**) VCTQ-MA containing PABC spacer and SVCTQ-MA without PABC spacer. All error bars shown are the SD from triplicate analyses. *n* = 3 per time point. LC-MS/MS technical replicates = 3. Monomer image and final figure created with Biorender.com.

The stability of the valine-citrulline linker in ADCs has been previously shown to be an issue with IV administration due to carboxylesterase cleavage in mouse blood (*44*, *47*). We found that removing the self-immolative para-aminobenzyl carbamate(PABC) linker segment resulted in a polymer prodrug that was more stable in blood by both IV and SC administration. This design may prove useful in other polymer prodrug applications as well where payload release in systemic circulation is undesirable. TQ was directly conjugated to the citrulline carboxyl group. The synthetic route for this blood-stable prodrug monomer SVCTQ methacrylate (referred to as SVCTQ-MA) can be found in fig. S1. The structural comparison between SVCTQ-MA and the original PABC-containing monomer (referred to as VCTQ-MA) can be found in Fig. 2E. The GalNAc ethyl methacrylate monomer (GalNAc-MA) for hepatocyte targeting was synthesized as previously described (*46*). Detailed monomer synthetic procedures and spectra can be found in the Supplementary Materials and figs. S1 to S5. Reversible addition-fragmentation chain-transfer (RAFT) polymerization (*53*) was used to synthesize the liver-targeted polymer prodrugs p(GalNAc-MA-co-SVCTQ-MA) and p(GalNAc-MA-co-VCTQ-MA), which will be referred to as pSVCTQ and pVCTQ, respectively. The synthetic scheme and results are summarized in Table 1. All polymerizations were well controlled with relatively narrow molecular weight distributions and a final average *M*_w_ of roughly 14,000 and 13,700 g/mol for pVCTQ and pSVCTQ, respectively. The experimental drug weight percent (drug wt %) were in close agreement with the targeted drug wt % in all polymerizations, with excellent batch-to-batch reproducibility. The polymer characterization results are described in the Supplementary Materials and figs. S6 to S9.

**Table 1.** Polymer synthesis and characterization summary. [M], monomer concentration; [CTA], chain transfer agent concentration; [I], initiator concentration; DP, degree of polymerization; *M*_n_, number average molecular weight; Đ, polymer dispersity.

Polymers	[M]:[CTA]:[I]	Monomer conversion	Theoretical DP of monomers	*M*_w_ (*M*_n_, g/mol)*	Đ^†^	Drug (weight %)
pVCTQ	38:1:0.13	87%	VCTQ-MA (4) GalNAc-MA (29)	14,000	1.1	14%
pSVCTQ	39:1:0.14	84%	SVCTQ-MA (4) GalNAc-MA (29)	13,700	1.1	15%

*Calculated by NMR spectroscopy based on monomer conversion and targeted DP.

†Determined by organic and aqueous SEC.

### Tissue pharmacokinetic studies demonstrate that pSVCTQ has higher liver:plasma TQ selectivity and exposures compared to oral TQ

The stabilized pSVCTQ design showed great improvements in liver:plasma TQ drug exposures, with an unexpected increase in absolute liver exposure and a greatly reduced *C*_max_ in plasma. The liver and plasma exposures of TQ were first compared after a single 25 mg/kg IV dose of pSVCTQ versus pVCTQ in mice to determine the benefit of the stabilized prodrug linker design (Fig. 2A). The blood stability was found to be notably different, where pSVCTQ greatly reduced TQ exposure in plasma, resulting in an improved liver:plasma area under the curve (AUC) ratio (Table 2). The linker that was devoid of the PABC unit led to reduced early release of TQ in the blood along with a lower *C*_max_ and AUC, consistent with abrogation of carboxylesterase cleavage (Fig. 2A). The prodrug designs exhibited similar PK profiles in the liver, including *C*_max_ and AUC values, demonstrating their excellent intracellular cleavage in this GalNAc/prodrug copolymer format.

**Table 2.** Summary of pharmacokinetic studies. RoA, route of administration; IV, intravenous; SC, subcutaneous; OG, oral gavage; AUC, area under curve from *t* = 0 to *t* = 336 hours; *C*_max_, maximum TQ concentration; *T*_max_, time at which maximum TQ concentration is achieved. p(GalNAc-MA-co-SVCTQ-MA) (pSVCTQ); p(GalNAc-MA-co-VCTQ-MA) (pVCTQ).

Test article	Dose (mg/kg)	RoA	Tissue	AUC (μg/g liver*hour or μg/ml plasma* hour)	AUC SD%	*C*_max_ (μg/g liver or μg/ml plasma)	*C*_max_ SD%	*T*_max_ (hour)	Liver:plasma AUC ratio
pVCTQ	25	IV	Liver	2725	7	48	5	8	120
			Plasma	22.62	16	0.88	22	2	
pSVCTQ	25	IV	Liver	2550	15	57	29	8	164
			Plasma	15.53	10	0.20	18	2	
pSVCTQ	10	SC	Liver	2422	13	25	13	8	252
			Plasma	9.617	8	0.10	2	24	
Parent TQ	10	OG	Liver	1404	20	17	23	8	62.8
			Plasma	22.35	14	0.38	12	8	

The optimized pSVCTQ design was selected for analysis by the SC administration route (Fig. 2B). pSVCTQ exhibited an unexpectedly higher TQ drug exposure in the liver compared to the oral TQ, demonstrating the GalNAc targeting effectiveness from the SC route of administration. The AUC values in the liver for pSVCTQ versus oral TQ were 2422 and 1404 μg/g*hour, respectively. The liver-targeted prodrug design via SC administration was also validated relative to oral drug blood exposure, with an AUC value of 9.617 μg/ml*hour for pSVCTQ versus 22.35 μg/ml*hour for the oral TQ. The *C*_max_ improvement was also much lower with 0.10 versus 0.38 μg/ml, respectively. Thus, there was an exciting improvement in tissue PK selectivity, with a 4-fold better liver:plasma AUC ratio (Table 2) and 3.8-fold lower plasma *C*_max_ for pSVCTQ compared to the oral drug.

TQ metabolism is known to be required for activity and is also responsible for hemotoxicity. Metabolites have been partially characterized previously, although the correlates of specific metabolite signatures for efficacy versus hemotoxicity are unfortunately not yet clearly available. Two key metabolites are carboxy-TQ (CTQ) and 5,6 orthoquinone-TQ (OQTQ), which have been previously characterized as markers of TQ metabolism in liver and blood (*38*, *54*–*59*). Both metabolites were found in liver at greater concentrations compared to plasma, with CTQ absence and low OQTQ in the plasma (fig. S11). This metabolite pattern thus follows prior results for the oral TQ PK and metabolism in mouse models with low OQTQ concentration in plasma (*55*, *56*). The urine profile was also characterized, and a high level of OQTQ metabolite was found after pSVCTQ SC administration, consistent with a urine signature for this metabolite reported previously in human trials involving TQ (*55*).

### Polymeric prodrug achieves high liver distribution and cross-liver hepatocyte intracellular delivery from the SC administration route

The *P. vivax* hypnozoites can develop in any liver hepatocyte and, upon reactivation, rapidly expand into symptomatic malarial disease from small liver numbers. TQ prodrugs must therefore achieve cross-liver hepatocyte delivery comparable to the liver exposure resulting from first-pass clearance of orally administered TQ. The liver distribution and intracellular hepatocyte delivery of pSVCTQ were thus characterized by organ-level IVIS imaging and at the cellular level by cross-sectional histology analysis (Fig. 2, C and D) following SC injection of rhodamine-labeled pSVCTQ [a description of the synthesis can be found in the Supplementary Materials and nuclear magnetic resonance (NMR) results in fig. S10] at the *T*_*max*_ of pSVCTQ (8 hours). pSVCTQ was found to accumulate in the liver with high selectivity at 8 hours after SC injection, with little fluorescence intensity observed in lungs, heart, spleen, and brain. A low level of fluorescence was observed in the kidneys, likely due to renal clearance of pSVCTQ.

Cross-sectional histology revealed nearly homogeneous liver penetration, with labeled polymer present in every hepatocyte (Fig. 2D). Little to no staining was seen in the sinusoidal lumen areas where Kupffer cells reside, consistent with ASGPR-targeted uptake specifically into hepatocytes. In the larger cross-sectional field, the polymer can be seen distributed across the liver rather than only in hepatocytes near the sinusoidal blood supplies. These targeting properties were hypothesized given the validated targeting activities of the FDA-approved siRNA therapeutic products (*60*, *61*), and these results validate the GalNAc targeting design of the chemically distinct pSVCTQ, showing excellent distribution to liver and hepatocytes specifically, and provide the targeting mechanistic validation that underpins the ability of this SC administered prodrug to provide efficacy comparable and greater than the oral drug standard that does achieve high liver localization by first-pass mechanisms.

### Polymeric prodrug has greater efficacy in the *P. berghei* luciferase and blood parasitemia model

**Fig. 3.**
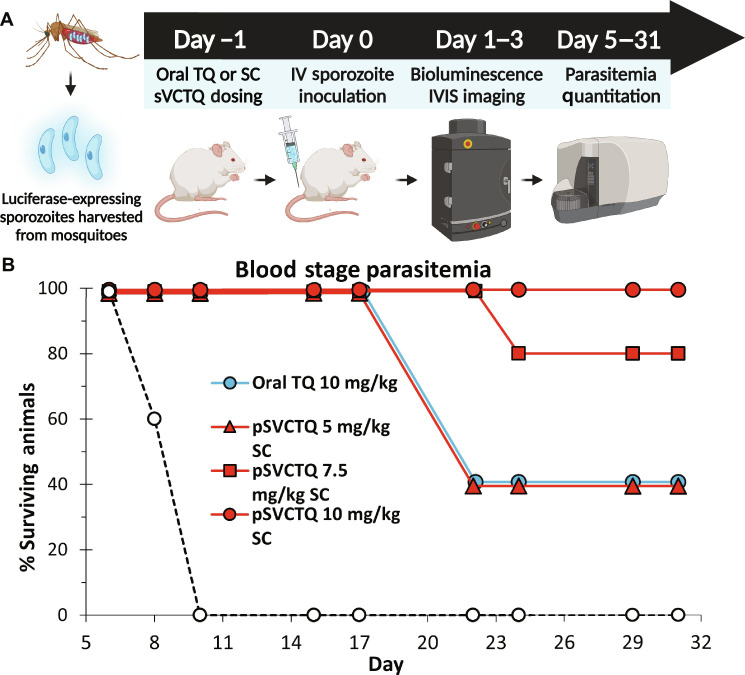
pSVCTQ has approximately twice the activity of oral TQ in the prophylactic *P. berghei* model. (**A**) Schematic of study design for the preexposure prophylactic model. Luciferase-expressing sporozoites are harvested from mosquitos. Mice are treated with either oral TQ or SC pSVCTQ and then inoculated with sporozoites. Bioluminescence of the sporozoites is recorded daily for 3 days using IVIS. From days 5 to 31, parasitemia is quantified using flow cytometry. (**B**) Results of the preexposure prophylactic dose-response study. Mice were dosed with either 10 mg/kg oral TQ or 5, 7.5, or 10 mg/kg TQ equivalent of pSVCTQ. No mice survived in the vehicle group. All mice survived in pSVCTQ 10 mg/kg SC group. *n* = 5 mice per treatment group. Schematic and final figure created with Biorender.com.

The *P. berghei* luciferase and blood parasitemia model has been used extensively to characterize TQ activity and to screen for alternative 8-AQ drug candidates (Fig. 3A) (*62*, *63*). *P. vivax* does not infect rodent models, but a model of acute infection using *P. berghei* that monitors liver sterilization of sporozoites has proven useful for evaluation of new antimalarial drug candidates, and, accordingly, the model has become an industry standard for evaluation of compounds with hypnozoiticidal potential. pSVCTQ was found to exhibit approximately twofold higher activity compared to oral TQ in this model (based on the TQ equivalent dose). The 24- and 48-hour whole-body IVIS imaging represents the initial homing to and expansion in the liver of the luciferase-expressing sporozoites, and thus is a measure of drug exposure and activity in the liver. By 72 hours, the liver-stage schizonts give rise to erythrocytic parasites, and thus, the 72-hour imaging time point represents both the liver and early blood stages (*62*). Both pSVCTQ and the oral TQ treatments completely suppressed the IVIS signal during days 1 to 3 at maximum doses of 10 mg/kg (fig. S13A). Subsequent blood parasitemia is the most sensitive measure of drug activity against the liver-based parasite, as even low surviving numbers can eventually give rise to full-blown blood parasitemia, requiring euthanasia (fig. S13B). pSVCTQ showed dose-dependent activity, with complete parasite suppression at 10 mg/kg (i.e., 100% causal prophylaxis). pSVCTQ at 5 mg/kg and oral TQ at 10 mg/kg showed comparable activity and partial suppression of parasitemia (Fig. 3B). The higher activity of pSVCTQ versus oral TQ in the *P. berghei* model thus mirrors the higher liver exposure achieved with polymer prodrug, demonstrating an important PK/PD mechanistic connection.

### The reduced plasma *C*_*max*_ and AUC TQ exposures of pSVCTQ result in a significant reduction in hemotoxicity compared to oral TQ

**Fig. 4.**
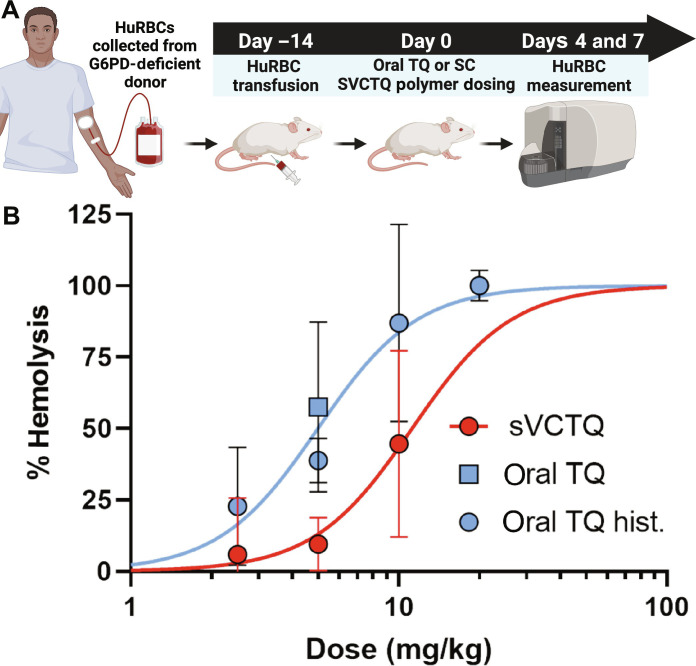
pSVCTQ reduces hemolytic toxicity in the humanized G6PD deficiency mouse model. (**A**) Schematic depicting study design of in vivo hemolytic toxicity study. RBCs are collected from a G6PD-deficient donor (huRBCs) and then engrafted into NOD/SCID mice. Mice with >60% huRBCs at the end of the transfusion period (day 0) are dosed with varying concentrations of oral TQ or SC pSVCTQ polymer solution. (**B**) Dose-response comparison of oral TQ with SC pSVCTQ in the huRBC engrafted NOD/SCID hemolytic anemia model. pSVCTQ and TQ titration data are from a single experiment each. Data for 5 mg/kg oral TQ, which served as the positive control across studies, are from multiple experiments. The percent hemolysis was calculated for each treated animal from the percentage of huRBC present on days 0 and 7, followed by normalization to the vehicle control group. The normalized data were fit with a two-parameter “agonist versus normalized response” curve fit with shared Hill slope, using GraphPad Prism software. The doses resulting in 50% hemolysis (TD_50_) and the 95% confidence intervals were 5.0 mg/kg (3.9 to 6.2) for oral TQ and 11.2 mg/kg (8.4 to 17.8) for pSVCTQ. An *F*-test analysis comparing the two data plots resulted in a *P* value for significance <0.0001. Vehicle, *n* = 3; all other groups, *n* = 4. Experiment was replicated three times. Oral TQ hist. indicates historical data for a hemotoxicity study using an oral dose of TQ. Schematic and final figure created with Biorender.com.

The humanized mouse model of G6PD deficiency is the industry standard for assessing the hemolytic anemia potential of 8-AQs owing to the use of fresh human RBCs (huRBCs) from G6PD-deficient donors (Fig. 4A) (*64*, *65*). The huRBCs for this experiment were from an African variant (A−) G6PD–deficient donor (2.3 U/g Hgb), which falls under class B per the G6PD variant WHO classification (*66*). Replicates of this study had G6PD enzyme activity between 0.5 and 3.1 U/g Hgb. The hemolytic toxicity of pSVCTQ was characterized after single SC doses of 2.5, 5, or 10 mg/kg. Oral TQ was dosed at 5 mg/kg to bridge historical TQ data in this model. The dose-response curves for pSVCTQ and oral TQ are shown in Fig. 4B. The median effective dose values, the dose that results in 50% huRBC loss by day 7 relative to vehicle control, were 11.4 and 4.7 mg/kg for prodrug and parent drug, respectively, translating into a greater than twofold reduction of hemotoxicity for pSVCTQ. Secondary measures of hemolytic anemia, including reticulocyte count, hematocrit, and spleen weight, correlated well with the huRBC results (fig. S14).

### Polymer prodrug shows anti-hypnozoite activity in an in vitro hepatocyte assay

**Fig. 5.**
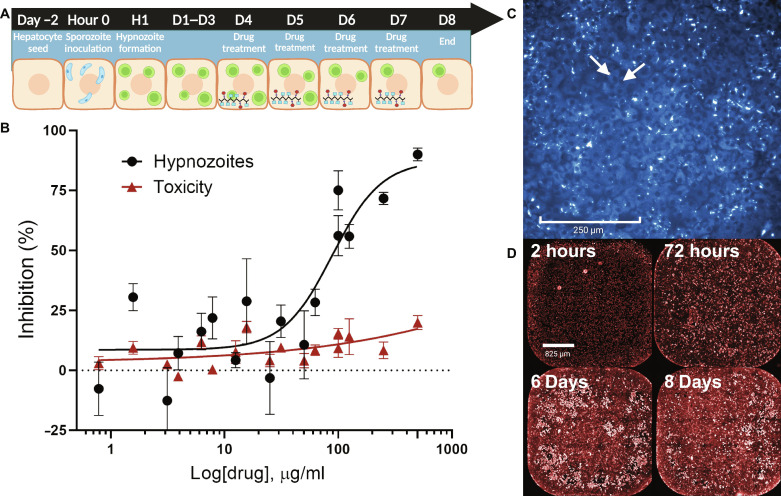
pSVCTQ shows anti-hypnozoite activity in the *P. cynomolgi*/rhesus primary liver cell model. (**A**) Schematic of study design for anti-hypnozoite assay. Plate wells are seeded with hepatocytes on day −2. On hour 0, the cells are infected with *P. cynomolgi* sporozoites from *Anopheles dirus* salivary glands. Time is allowed for hypnozoite formation, and cells are treated with pSVCTQ solution on days 4 to 7. On day 8, the cells are fixed and stained for imaging and hypnozoite detection. (**B**) Anti-hypnozoite activity of pSVCTQ and toxicity to hepatocytes. (**C**) Free TQ is visualized inside cells when imaged at 355 nm following release from pSVCTQ. Cells were bathed in a solution of 500 μg polymer/ml PBS. Arrows indicate cell nucleus. (**D**) Representative images of ASGPR expression time course. Fixed cells were visualized using human ASGPR1 Alexa Fluor 647–conjugated antibody and then imaged at 647 nm. A steady increase of ASGPR is seen up to day 6 and then a slight decrease on day 8. The study was replicated twice. Schematic and final figure created with Biorender.com.

The new *P. cynomolgi* anti-hypnozoite drug screening platform was used to characterize the activity of pSVCTQ against hypnozoites (*52*). *P. vivax* only infects NHP and humans, and NHP cost and current availability constraints would limit group sizes to single doses, sufficient only for a binary readout on efficacy. Since the TQ payload is already well established to have hypnozoiticidal activity, primate studies were ruled out for cost and ethical concerns. Nevertheless, it was of interest to be able to gauge potency against hypnozoites, which could guide future structure-activity relationship work around GalNAc placement, density, and valency, for example. The *P. cynomolgi* anti-hypnozoite drug screening platform has been used to characterize the activity of the pSVCTQ polymeric prodrug against hypnozoites (*52*). NHP hepatocytes were maintained in a culture system and infected with *P. cynomolgi* sporozoites, and then incubated with polymer solutions of varying concentrations to capture the anti-hypnozoite activity [hypnozoite median inhibitory concentration (IC_50_)] and hepatocyte toxicity (hepatocyte IC_50_). pSVCTQ showed a hypnozoite IC_50_ of 25.6 μM TQ and a hepatocyte IC_50_ at greater than 161 μM TQ. While hepatocyte cell culture platforms do not recapitulate physiological ASGPR expression levels, we have shown that the cell-impermeable pSVCTQ does still target receptor-mediated endocytosis in this ex vivo model. The TQ released inside the *P. cynomolgi–*infected hepatocytes achieved potent anti-hypnozoite activity (Fig. 5). pSVCTQ cannot be compared directly in dose response to parent TQ, as the TQ lipophilicity and permeability allows it to rapidly enter cultured cells (*67*), while pSVCTQ is dependent on receptor-mediated endocytosis and the ASGPR expression levels that are lower and vary over time in the cultured hepatocytes.

### COGS and manufacturability analysis shows feasibility of mass-produced polymeric prodrug

We have conducted an initial COGS and manufacturability analysis to look at the rollout feasibility of this polymeric prodrug product. A second key goal was to document how the polymeric prodrug design could be optimized to address the global health and health inequity issues associated with COGS in disease spaces such as malaria. We found that polymeric prodrug redesigns could substantially reduce the projected COGS, which may be of relevance to polymeric prodrugs more generally since drug costs can be an issue in economically developed countries where health insurance inequities exist. 

The exact scale and timing of polymeric prodrug production required for a mass administration eradication campaign is uncertain given different burden estimates and rollout challenges across so many countries (*2*, *3*). We first selected a manufacturing scale of 5 million doses given the WHO estimate of annual *P. vivax* infections. The analysis suggested that the monomer and RAFT chain transfer agent (CTA) could be made non–Good Manufacturing Practice (GMP) under international quality standards. The one-step RAFT polymerization of the final polymeric prodrug would then be conducted under GMP processes. No unusually hazardous or reactive materials and synthetic conditions were needed for the CTA, monomer building blocks, and the final polymeric prodrug. The equipment footprint requires conventional and available reactor scale, and purification equipment that is already established with contract manufacturers. At full manufacturing production, the campaigns could be optimized with shorter time periods and larger reactor scale 800-2500L reactor equipment.

COGS estimates were based on a plant time model ($/day of operation, factoring in equipment scale), including labor, utilities, depreciation, laboratory direct and indirect costs, and contractor profit. The analysis assumed manufacturing at a low-cost facility (e.g., India or China) but still meeting international quality standards appropriate to each step (non-GMP and GMP). We first conducted an analysis of the primary copolymer design, with one compositional optimization of using a Val-Ala linker that gave similar PK performance (fig. S15). This prodrug monomer with the Val-Ala linker provides synthetic chemistry advantages, as the SVATQ monomer is more soluble in dimethyl sulfoxide (DMSO) and requires lower quantities of the total solvent. The more homogeneous polymerization solution also makes monomer conversion and final compositional identity more reliable batch to batch.

The corresponding COGS raw material and processing costs are reported in Table 3 for the Val-Ala copolymer design at 5MM doses. The comonomer ratios and degree of polymerization (DP)/*M*_w_ were kept the same as pSVCTQ. The final estimated COGS for this polymeric prodrug with the copolymer design was $2.02 per dose (1-g polymeric prodrug cost without vialing or syringing).

**Table 3.** COGS raw materials and processing estimates for copolymer design at 5MM doses.

Product	Production Mkg/year	Raw materials $MM	Processing $MM	Total $MM	Unit cost $/kg
GalNAc monomer	4.36	4.30	1.40	5.71	1309
Prodrug monomer	1.52	2.62*	1.20	3.82	2505
Product polymeric prodrug	6.0	9.62^†^	0.50	10.10	1683

*TQ drug component estimated at $2000/kg.

†Includes the total cost to produce the three components above.

This analysis suggested that further COGS design pressures could improve the cost per dose without affecting its pharmaceutical functionality. The first alternative ter-copolymer design blended in a lower-cost solubilizing monomer to substitute for some of the GalNAc targeting monomer. The 2-(methylsulfinyl)ethyl methacrylate (MSEMA) monomer was chosen because of its excellent water solubilities (*68*, *69*). The ter-copolymer design is due to the lower cost of the MSEMA monomer at $261/kg compared to the GalNAc monomer at $1309/kg. The COGS analysis was performed at increasing MSEMA monomer incorporation with 10% corresponding to $1.93 per dose, with 25% corresponding to $1.75 per dose, and at 50% corresponding to $1.55 per dose (compared to the copolymer design above at $2.02 per dose). We have shown that a lower GalNAc polymeric prodrug design preserved much of the PK properties (fig. S15), and further optimization could likely achieve similar PK properties.

**Fig. 6.**
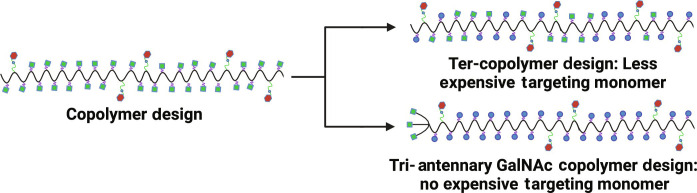
Engineering liver-targeted polymeric prodrug design for lower COGS. Two designs were characterized and shown to reduce projected COGS while maintaining pharmaceutic functionality. In the first design, the copolymer was changed to a ter-copolymer design to substitute the more costly GalNAc targeting monomer with a less costly solubilizing monomer. The second design completely replaced the GalNAc targeting monomer, with the targeting ligand introduced via a tri-antennary GalNAc CTA. Created with Biorender.com.

This analysis shows that considerable COGS price reduction can be achieved by premeditated focus on polymeric prodrug composition and designs (Fig. 6) that reduce more costly monomer components. In this second design, a tri-antennary GalNAc CTA was incorporated to introduce the targeting ligand that has been validated in the FDA-approved siRNA drug product (*70*). The costly GalNAc monomer was thus completely removed. We additionally wanted to look at a broader range of production levels, since radical cure campaigns may eventually require more doses than the 1-year case numbers. The COGS parameters were estimated then at a large global scale of 50MM doses as summarized in Table 4. The COGS was determined to be a promising $1.27/g dose at this higher manufacturing scale. We are separately reporting a more complete analysis of polymeric linker and targeting designs, and here, we show that a tri-antennary GalNAc polymeric prodrug was equivalent to the copolymer in liver AUC drug exposure (fig. S15).

**Table 4.** COGS estimates for tri-antennary GalNAc design at 50MM doses.

Product	Production Mkg/year	R.M. $MM	Proc. $MM	Total $MM	Unit cost $/kg
Tri-antennary GalNAc CTA	6.60	2.57	2.15	4.72	715
Prodrug monomer	15.2	20.3*	5.00	25.3	1664
MSEMA	38.2	4.60	1.20	5.8	151
Product polymeric prodrug	60.0	36.4^†^	1.40	37.8	630

*TQ component estimated at $2000/kg is $2.43MM/year.

†Includes the total cost to produce the three components above.

This design thus achieves a substantial 63% reduction in COGS per dose, showing how cost pressures can be lowered with the goal of increasing patient accessibility in the many places throughout the world that face drug cost, acquisition, and health insurance inequities. We have also then performed an estimate of how overall doses per year affect the COGS, as successful drug campaigns might reduce the caseload over time. The cost per dose of this design is estimated to rise to $1.40 at 5MM doses, to $2.64 at 1MM doses, and to $4.84 at 0.1MM doses. The overall cost goes down as the doses needed are reduced, even as the individual dose cost goes up at a lower manufacturing scale. In perspective of malaria impacts on humans and human societies, the total costs are quite reasonable. These results show how the polymer could be engineered and optimized for COGS requirements and health equity, rather than by the therapeutic index alone.

## DISCUSSION

TQ’s impact as a new radical cure drug agent against *P. vivax* malaria has been constrained in part by the risk of hemolytic toxicity in G6PD-deficient human populations that are largely coincident with the malaria belt geography (*23*, *25*, *71*, *72*). TQ requires only a single oral dose, compared to the only prior radical cure drug PQ that requires 14 daily doses. However, TQ’s hemolytic toxicity, along with other potential issues of dosing level and availability of the concomitant chloroquine or dihydroxy artemisinin co-therapy (*73*–*75*), has so far prevented its widespread implementation. Here, we have validated an SC administered and liver-targeted TQ prodrug design that is aimed at achieving a higher therapeutic index sufficient to enable mass administration without G6PD testing. Mechanistically, the change in taking TQ on this hepatocyte-targeted delivery route could lower the dose needed for radical cure and/or favorably alter drug metabolism from gastrointestinal (GI) and first-pass metabolism. A clinical study involving heterozygous female volunteers with the G6PD Mahidol variant showed that this population did not exhibit the study’s hemotoxicity definition with a threefold lower 100-mg oral TQ dose. While heterogeneity in G6PD deficiency levels across human populations make the exact therapeutic index requirement uncertain, a minimal requirement is probably thus at least three- to fivefold and may be higher in some regions where more severe G6PD deficiency is found. TQ drugs with therapeutic index increases could also prove relevant as new point-of-care diagnostic tools can enable at least some swaths of G6PD-deficient patients to be treated by defining enzyme deficiency cutoff values. The combination of inexpensive point-of-care diagnostics, population modeling, and higher therapeutic index drugs could knock down infections to a point where significant mass eradication becomes a reality (*25*).

We have shown that a single SC dose can achieve a fourfold increase in liver:blood PK AUC ratio compared to the oral TQ equivalent. Recent studies have begun to show the potential of polymer-drug conjugates given by the SC route of administration (*76*–*79*). This work demonstrates that a polymeric prodrug with an enzyme-cleavable linker can function effectively through the SC route. This may open other liver and organ therapeutic applications benefiting from the blood/lymphatic stability and intracellular release properties. The PABC self-immolative linker segment removal was critical to reducing mouse carboxylesterase cleavage and subsequent blood exposure of TQ via the SC route (*80*–*84*). This pSVCTQ design reduced the *C*_*max*_ of TQ in blood by over 75% and the blood AUC over 14 days by more than 50%. It reduces hemotoxicity in the humanized G6PD deficiency mouse model by the corresponding two- to threefold reduction in TQ blood exposure. The altered linker still allows for highly efficient cleavage of TQ in liver hepatocytes, as shown by the rapid appearance of the free TQ drug in the liver PK measurements (*C*_*max*_*/T*_*max*_ at 8 hours). Additional linker and polymer designs that slow the TQ release in hepatocytes could further reduce hemotoxicity by lowering the amount of drug recirculating into the blood. We have also characterized two key 8-AQ metabolites that have been extensively characterized with PQ in the past and to a lesser extent with TQ (*38*, *54*–*59*, *85*). The CTQ and OQTQ were not found at significant levels in the plasma, while they were both found in the liver. It was notable as well that the OQTQ was found at a relatively high level in mouse urine, consistent with Cyp2D metabolism as reported for oral TQ in humans (*55*). Together, these PK/PD studies in efficacy and hemotoxicity suggest that the liver-targeted TQ given by the SC route enhance activity and reduce hemotoxicity by altering blood bioavailability and the liver biodistribution.

Although TQ metabolism is known to be required for anti-hypnozoite activity, the correlates and biomarkers of TQ metabolites as they relate to efficacy versus hemotoxicity in humans or animals remain poorly defined. These initial results across the PK, efficacy, and hemotoxicity rodent models are promising, but a limitation in radical cure therapy development is the lack of NHP models for *P. vivax* anti-hypnozoite activity. Although *P. cynomolgi* is a relapsing parasite that grows in the rhesus macaque (*Macaca mulatta*), it is separate and distinct phylogenetically from *P. vivax* that infects humans (*86*, *87*). The TQ concentration-response relationships as a function of dose and time are quantitatively different, given species differences in absorption, metabolism, transporters, distribution, and excretion, making it difficult to directly translate from this model to humans with TQ and TQ prodrugs. Although physiological-based pharmacokinetic (PBPK) modeling can help with this translation, special consideration would need to be given to tissue partition coefficients in the development of a full PBPK model, especially since 8-AQs are in higher concentrations in the liver relative to plasma in the *P. cynomolgi* animal model (*56*, *62*, *88*, *89*). PBPK modeling from these combined rodent models could therefore justify taking these SC polymeric prodrugs into human experimental medicine directly, rather than testing in NHP models.

Another key consideration in the target candidate profile for radical cure drugs is that new candidates for mass administration must meet the single-dose requirement. New oral drugs would be preferred over SC administered drugs in terms of ease-of-use in global health settings, but it has proven difficult to find new 8-AQs or other oral small-molecule drugs that are effective against the metabolically inactive hypnozoites (*40*, *41*, *90*). The COGS and manufacturability analysis provide evidence that this polymeric prodrug design could meet the cost requirements for global eradication campaigns in resource-limited countries. An SC drug would require a higher level of caregiver training and costs that go beyond the COGS analysis. The coronavirus disease vaccines offer a favorable precedent for such a vialed SC product given at high patient scale in many places around the world and a cautionary tale of uptake inequality for many disadvantaged and historically oppressed patient populations globally. There are also formulation issues to be addressed relating to the SC volume and dose, given the 300-mg or higher dose of TQ needed for human radical cure (*37*, *38*). There are FDA-approved therapeutics with high SC dosing volumes, such as Sunlenca given as 2 × 1.5-ml injections or Vidaza with up to 4-ml SC injection (*91*–*94*). To minimize the dosing volume while achieving adequate drug exposure, the drug weight percent in the polymeric prodrug would need to be optimized, along with the solubilizing comonomer in the context of an optimized formulation solvent.

In summary, we have demonstrated here that single SC administrations of the liver-targeted polymeric TQ prodrug can achieve substantial increases in tissue PK blood:liver selectivity and improve corresponding efficacy to hemotoxicity ratios in key radical cure drug development animal models. The polymeric prodrug with the blood-stabilized linker showed that an SC route of administration can ameliorate the burst release of drug in plasma while targeting and improving liver drug exposure. The latter was unexpected in finding that the liver exposure could be improved by this alternative delivery route and with the GalNAc targeting of liver hepatocytes. This demonstrates that SC administered polymeric prodrugs incorporating the GalNAc targeting ligand can efficiently traffic and target hepatocyte uptake in a similar manner to the clinically validated route for siRNA delivery (*60*, *70*, *95*, *96*). Targeting biology studies first have demonstrated that the SC administered pSVCTQ is selectively targeting the liver, with the GalNAc targeting ligand reaching nearly all the intracellular compartments of hepatocytes where hypnozoites might reside. PK/PD studies have quantitatively shown a close correlation between increases in blood:liver TQ drug exposure selectivity and activity versus hemotoxicity relationships in a key *P. berghei* efficacy model versus the humanized G6PD deficiency mouse model. Last, the current state-of-the-art liver-stage hypnozoite model and drug screening platform (*52*) has been used to demonstrate the dose-dependent anti-hypnozoite activity of pSVCTQ. While the ASGPR expression levels of the primary NHP hepatocytes are variable over time, and thus the IC_50_ values of the pSVCTQ released drug cannot be compared directly to the cell-permeable TQ, it is clear that pSVCTQ has excellent anti-hypnozoite activity in this important drug screening platform (*52*). The fully synthetic manufacturing footprint (*46*, *97*) was found to offer promising cost of goods per dose and manufacturability with existing small molecule and RAFT polymer reactor chemistry footprints. Together, these results validate the liver-targeted TQ prodrug design platform as an important therapeutic approach to the spectacularly unmet need for radical cure malaria therapeutics.

## MATERIALS AND METHODS

### Study design

This study was designed to identify a lead polymeric prodrug and describe the PK, activity, and hemotoxicity of this polymer in a controlled laboratory environment. pSVCTQ was hypothesized to have improved PK distribution, increased activity, and reduced hemotoxicity over oral TQ administration. This was done through in vivo mouse studies and an in vitro hepatocyte assay.

For PK studies, power calculations were made to select the sample size, and this number is validated in historical work (*46*). For the prophylactic activity study, a sample size of five was necessary on the basis of a power analysis. The hemotoxicity study had a minimum sample size of four based on power calculations and validated in historical data (*64*); however, the vehicle control group had an *n* of 3 based on historical study findings. During the early engraftment period of huRBCs in mice, some will not meet the predetermined criteria to be considered for the dosing study. There were not enough mice to have a sample size of four for all groups, so the vehicle control group was selected to have one less mouse due to the stability of huRBC in that group. An overabundance of mice could not receive huRBC engraftments to make up for animals that do not meet the study criteria due to the difficulty of receiving a substantial amount of G6PD-deficient blood from the same donor. This scarcity is why a fourth high-dose polymer group could not be included in this experiment.

Samples were not randomized or blinded. The PK experiments were run four separate times using the same polymer batch, and data presented here are representative of these studies. Liquid chromatography tandem mass spectrometry (LC-MS/MS) analyses were replicated twice from the same experimental samples under the same conditions to ensure consistency between runs. Data for both oral TQ and pSVCTQ PK are from the same experiments. The prophylactic activity and hemotoxicity studies were performed thrice showing general improvement in comparing TQ and pSVCTQ; the data presented here are representative of that improvement while highlighting a dose response. The in vitro anti-hypnozoite assay was replicated twice under the same conditions to ensure consistency. End points were selected prospectively for all studies. There were no animal exclusion criteria for the PK studies and prophylactic activity experiment, and all data points are included. For the hemotoxicity experiment, mice were excluded from the analysis if there were inconsistencies with the percent huRBC and mouse reticulocytes levels on days 4 and 7 when compared to trends from previous hemolytic toxicity experiments. Hemotoxicity experiments were excluded if the huRBC engraftment was below 70% or if the G6PD activity was below U/g Hgb due to the fragility of the huRBCs.

### Reagents

Chemicals and reagents were purchased from Sigma-Aldrich (St. Louis, MO) unless otherwise specified. GalNAc ethyl methacrylate (GalNAc-MA) monomer, 5,6 orthoquinone TQ, and CTQ were purchased from Medchem Source (Federal Way, WA). 4-Cyano-4-(ethylsulfanylthiocarbonyl) sulfanylpentanoic acid (ECT) was purchased from Omm Scientific (Dallas, TX). TQ was purchased from MedKoo Biosciences (Morrisville, NC). Methanol, ultrapure H_2_O, Dulbecco’s phosphate-buffered saline (DPBS), heparin-coated tubes (BD), DAPI (4′,6-diamidino-2-phenylindole), and Alexa Fluor 488 phalloidin were purchased from Thermo Fisher Scientific (Waltham, MA). Sephadex G-25 prepacked PD10 columns were obtained from GE Healthcare Life Sciences (Pittsburg, PA). High-performance liquid chromatography (HPLC)–grade methanol and water were purchased from VWR International Ltd. (Radnor, PA).

### Synthesis of TQ prodrug monomers

The synthetic scheme illustrated in fig. S1 was followed to obtain the prodrug monomer SVCTQ-MA carrying enzyme labile linker valine-citrulline. All synthesized monomers and intermediates were purified by precipitation and/or silica gel column chromatography techniques. The successful synthesis and purity of the monomers were confirmed and characterized by NMR spectroscopy (Bruker Avance spectrometers 300 MHz) and electrospray ionization MS (ESI-MS; Bruker Esquire ion trap mass spectrometer). Detailed experimental procedures, NMR spectra, and ESI-MS spectral characterization of SVCTQ-MA are provided in the Supplementary Materials (figs. S2 to S5). The synthetic procedures for GalNAc-MA and VCTQ-MA have been previously published (*46*).

### Polymer synthesis

#### RAFT polymerization

In general, RAFT polymerization of GalNAcMA and prodrug monomer was conducted in DMSO-d6 under a nitrogen atmosphere using ECT as the CTA and V70 as the radical initiator. A *T*_0_ sample of the homogeneous solution was taken and stored at −20°C for the determination of monomer conversion. The reaction was then placed in a preheated oil bath at 42°C. After 22 hours, the reaction was stopped through the introduction of oxygen by removing the septa and cooling the solution with a mild stream of air. After the solution was cooled to ambient temperature, a *T*_final_ sample was taken and stored at −20°C for the determination of monomer conversion. The polymer was purified by precipitation followed by dialysis against DMSO and water. The lyophilized polymer was further purified by a PD10 desalting column. The theoretical molecular weight was calculated on the basis of the targeted DP and monomer conversion. The TQ drug wt % was calculated by ^1^H NMR spectroscopy using levofloxacin as an internal standard. More detailed experimental procedures, NMR spectrum, and gel permeation chromatography spectrum can be found in the Supplementary Materials.

#### Synthesis of rhodamine-labeled SVCTQ polymer

The rhodamine (RheMA)–labeled SVCTQ copolymer p(GalNAc-MA-co-SVCTQ-MA-co-RheMA) was synthesized and used for biodistribution and histological studies. pSVCTQ was synthesized using the same parameters as described above, only with 1 DP of rhodamine methacrylate added to the polymer design. Synthetic details can be found in the Supplementary Materials.

#### SEC and NMR spectroscopy

Polymer molecular weight distributions were determined using a Tosoh size exclusion chromatography (SEC) TSK-GEL α-3000 and α-e4000 columns (Tosoh Bioscience, Montgomeryville, PA) connected in series to an Agilent 1200 Series Liquid Chromatography System (Santa Clara, CA), Wyatt Technology miniDAWN TREOS, three-angle multiangle light scattering instrument, and Optilab TrEX, refractive index detector (Santa Barbara, CA). HPLC-grade *N*,*N′*-dimethylformamide containing 0.1 wt % LiBr at 60°C was used as the mobile phase at a flow rate of 1 ml/min in a nonaqueous SEC system. Absolute molecular weight averages (*M*_n_ and *M*_w_), polydispersity indices, and dn/dc were calculated using ASTRA software (Wyatt). Monomer conversion was determined by ^1^H NMR spectroscopy from the crude (*T*_0_ and *T*_final_) polymer solution. Molecular weight was determined on the basis of monomer conversion. The final polymer compositions and drug wt % of the purified and lyophilized polymers were determined using ^1^H NMR spectroscopy (Bruker Avance spectrometers 300 MHz).

### Pharmacokinetics, biodistribution, and subcellular hepatocyte localization studies

#### Animals

Female BALB/c mice (6 to 9 weeks; The Jackson Laboratory) were used and housed in a temperature- and humidity-controlled room with 12-hour light/dark cycles. Food and water were available ad libitum*.* All animal handling and experiments were performed according to methods approved by and in compliance with the University of Washington Institutional Animal Care and Use Committee and the Animal Use (IACUC).

#### Pharmacokinetic studies

In vivo PK analysis of the polymers was compared in mice. All mice were anesthetized with 3% isoflurane and O_2_ at 1 liter/min and then dosed retro-orbitally or SC with a solution equivalent to 10 to 25 mg/kg TQ in DPBS (volume of dosing solution of 5 μl/g of mouse). There were nine time points in total for each treatment group (*n* = 3). At the specified time points following dosing (30 m, 2 hours, 4 hours, 8 hours, 24 hours, 48 hours, 7 days, and 14 days), mice were anesthetized with isoflurane and whole blood was collected via cardiac puncture, which was followed by cervical dislocation. Blood was centrifuged at 2000*g*, 4°C for 10 min, and then plasma supernatant was collected. Urine was collected by placing a weigh boat under mice during exsanguination. Liver was collected fresh, weighed, mixed with equal mass of molecular biology–grade H_2_O, and then homogenized. All in vivo samples were stored at −80°C for LC-MS/MS analysis.

#### Polymer biodistribution imaging

Mice were dosed SC with rhodamine-labeled pSVCTQ at the dose of equivalent 25 mg/kg TQ, which were then euthanized at 8 hours after injection. Selected organs, including liver, kidneys, lungs, spleen, heart, and brain, were harvested and washed with PBS before imaging studies (IVIS) using fluorescent mode at (excitation/emission: 530/585 nm), Caliper Xenogen IVIS (PerkinElmer, Hopkinton, MA). The radiant efficiency emitted from the organs in each mouse was subsequently quantified using Xenogen Living Image software. Mice treated with PBS served as controls (fig. S12). Three mice were included in each group.

#### Liver hepatocyte targeting studies

Rhodamine-labeled pSVCTQ was formulated in PBS and SC administered in mice at a dose of 25 mg/kg TQ equivalent. Mice receiving PBS served as controls. The liver was harvested at 8 hours after injection and immediately fixed in 10% neutral buffered formalin at 4°C for 4 hours. After washing three times in cold PBS, the liver tissue was sunk in 15% sucrose solution for 24 hours and then in 30% sucrose solution for 24 hours at 4°C. The sucrose solution was prepared in DPBS (1×, with Mg^2+^ and Ca^2+^). The liver tissue was then blotted with paper tissue and embedded in an optimal cutting temperature compound. The sample was frozen in prechilled isopentane in liquid nitrogen and stored at −80°C. The 5-μm-thick sections were prepared using CryoStat at −20°C. Alexa Fluor 488 phalloidin was diluted 1:50 in PBS and used to counterstain the sections for 1 hour at room temperature (RT) to visualize cell outlines. The cell nuclei were counterstained with DAPI (blue) for 5 min at RT. Sections were imaged using an epi-fluorescent microscope (Nikon Eclipse 90i and Hamamatsu C10600 Camera at 20× magnification) using GFP-B HYQ (excitation/emission: 450/550 nm) Texas red HYQ (excitation/emission: 535/580 nm) and ultraviolet illumination (excitation/emission: 350/470 nm).

### LC-MS/MS analysis

TQ and its metabolites were extracted from liver homogenate, plasma, and urine samples before LC-MS analysis. This method for TQ quantification using LC-MS/MS has been previously described (*46*). Briefly, internal standards in a 1:1 water/methanol solution were added to the given matrix, diluted 1:10 with neat methanol, and vortexed. The supernatant containing the analytes was separated from the solid protein-containing pellet following 20 min of 4°C centrifugation at 17,500*g*. The supernatant was then further diluted with water to yield a 1:1 water/methanol tissue extract, which was subjected to centrifugation for 10 min at 17,500*g*, 4°C. This solution was then transferred to a low-recovery glass LC-MS vial for analysis.

Primary stock solutions of TQ, OQTQ, and CTQ were prepared at 5 mg/ml concentration in DMSO. The internal standard stocks for TQ-d3 were prepared at 5 mg/ml in DMSO. All primary DMSO stocks were stored at −20°C in amber glass vials. TQ assay calibrators were prepared in a completely analogous procedure to the one described above for the processing of study samples, with the calibrators added together with the internal standard to naive liver homogenate, plasma, or urine matrix. For analysis of TQ in liver samples, the calibrators were chosen to cover the concentration range between 80 μg/g liver homogenate and 20 ng/g liver homogenate. For analysis of TQ in plasma and urine, the calibrators were chosen to cover the concentration range between 2 and 1 ng/ml.

LC-MS/MS analysis of TQ concentrations was performed on the Waters XEVO TQS system at the UW School of Pharmacy facility. Following sample injection of 15 μl for plasma and urine, and 5 μl for liver, the analytes were resolved on a Poroshell 120 EC-C18 column (150 mm–by–2.1 mm internal diameter; 2.7-μm particle size) (Agilent Technologies) at RT using a gradient elution, with the mobile phase consisting of water (A) and methanol (B), each with 0.1% formic acid. The following 0.25 ml/min gradient was applied throughout: 0 to 1 min, maintained at 50% B, and then increased to 100% B from 1 to 5.5 min and held at 100% B from 5.5 to 8 min. The column was washed during each run by decreasing B from 100% to 10% over 2 min, holding at 10% B for 1 min, and an equilibration period of 4 min whereby B is increased to 20% in 0.1 min and held at 20% B for 3.9 min.

TQ was quantified by monitoring the peak area ratio of multiple reaction monitoring (MRM) transitions specific to TQ [464→379 mass/charge ratio (*m*/*z*)] and the internal standard d3-TQ (467→382 *m*/*z*). CTQ was quantified by monitoring the peak area ratio of MRM transitions specific to CTQ (481→379 *m*/*z*) and the internal standard d3-TQ (467→382 *m*/*z*). OQTQ was quantified by monitoring the peak area ratio of MRM transitions specific to OQTQ (303→219) and the internal standard d3-TQ (467→382 *m*/*z*). TQ and d3-TQ were eluted at 6.03 min, with the collision for the transitions specified above optimized to 15 eV. OQTQ was eluted at 2.39 min, with the collision energy optimized to 26 eV. CTQ was eluted at 7.93 min, with the collision energy for the above transition optimized to 25 eV.

### TQ drug activity in a *P. berghei* model

Efficacy studies in the *P. berghei* mouse model of causal prophylaxis were conducted at the Walter Reed Army Institute of Research, Experimental Therapeutics Branch as previously described (*46*).

#### Sporozoites

Luciferase-expressing *P. berghei* ANKA sporozoites were obtained from laboratory-reared female *Anopheles stephens* mosquitoes as previously described (*46*). Sporozoites were recovered by the method of Li *et al.* (*62*) and quantitated using a hemocytometer. Sporozoites isolated from the same batch of mosquitoes were inoculated into mice on the same day to control biological variability in sporozoite preparations. Each animal was inoculated IV in the tail vein with approximately 10,000 sporozoites suspended in 0.1-ml volume on day 0.

#### Animals

Age-matched, 40 young adult (7-week-old) female C57BL/6 albino mice were purchased from Charles River Laboratories (Frederick, MD). On arrival, animals were acclimated for 7 days in quarantine. Mice were housed and maintained at a temperature range of 64° to 79°F, 34 to 68% relative humidity, and a 12-hour light/dark cycle. Food and water were provided ad libitum. Animals were fed a standard rodent maintenance diet. All animal use, care, and handling was conducted under an IACUC-approved animal use protocol in an Association for Assessment and Accreditation for Laboratory Animal Care International (AAALACi)–accredited facility with a Public Health Services Animal Welfare Assurance and in compliance with the Animal Welfare Act and other federal statutes and regulations relating to laboratory animals.

#### Dosing of polymer therapeutics and parent TQ

TQ was dissolved at a stock concentration of 1 mg/ml in 25% hydroxy-β-cyclodextrin acidified to pH 5 with acetic acid and diluted in DPBS for final dosing solutions. Polymers for parenteral administration were prepared in DPBS at the final dosing concentrations. All drug concentrations were calculated per the free base molecular weights of TQ. TQ parent compounds were administered intragastrically via an intragastric feeder tube. Vehicle groups (0.1 ml of DPBS) and polymer groups were administered SC in the right flank. Free TQ, vehicle, and polymers were given as a single dose on day −1. In this model, the pSVCTQ doses studied were 5.0, 7.5, or 10 mg/kg TQ equivalent.

#### IVIS imaging studies of liver parasite suppression

Whole-body IVIS imaging of bioluminescence activity from luciferase-expressing *P. berghei–*infected mice was performed using a PerkinElmer IVIS Spectrum. Mice were evaluated at 24, 48, and 72 hours after sporozoite inoculation to determine liver- and blood-stage malaria infection. Mice received luciferin (150 mg/kg, Gold Biotechnology, St. Louis, MO) intraperitoneally in a maximal volume of 150 μl. Three minutes after luciferin administration, the mice were anesthetized with inhaled isoflurane and positioned ventral side up in the IVIS Spectrum on a 37°C platform. The mice continued to receive isoflurane through nose cone delivery. The camera exposure times used were 1 or 5 min for the 24-, 48-, and 72-hour time points with *f*-stop = 1, and the large binning setting was chosen. Quantitative analysis of bioluminescence emitted from whole bodies or region of intensity (ROI) were determined by measuring the luminescence signal intensity in photons per second using the ROI settings of the Living Image 3.0 software. The ROI, expressed in total flux of photons, was set to measure the abdominal area at the location of the liver from whole-body imaging (*62*). The data were generally found to fit a normal distribution. Means and SDs of photon measurement were calculated.

#### Parasitemia measurements

On days 5 to 31 after infection, mice were analyzed for blood-stage infections by quantitation of malaria parasites in erythrocytes by flow cytometry. Blood (3 μl) from the mouse tail was collected directly into 0.3 ml of 1% heparinized isotonic buffer (PBS saline). After addition of 1 ml of 0.04% of glutaraldehyde for fixation, the samples were incubated at 4°C for 60 min. Cells were centrifuged at 450*g* for 5 min. The supernatant was removed by aspiration, and the cells were resuspended in 0.5 ml of PBS buffer supplemented with 0.25% (v/v) Triton X-100 and kept at RT for 10 min. After centrifugation, the permeabilized cells were resuspended in 0.5 ml of ribonuclease at 1 mg/ml concentrations and incubated for at least 2 hours at 37°C to ensure complete digestion of reticulocyte RNA. YOYO-1 dye (from 1 mM stock solution in DMSO, as supplied by the manufacturer) was diluted to 2500 ng/ml in PBS. Then, 20 μl of the YOYO-1 solution in the dilution was added to 0.5 ml of sample for a final dye concentration of 100 ng/ml of YOYO-1, which was found to be optimal to discriminate infected erythrocytes from the lowest (0.01%) to the highest parasitemia counts (74.0%).

All flow cytometric analyses were carried out with an FC500 MPL flow cytometer (Beckman Coulter, Fullerton, CA) for conducting five-color analysis from either single or dual laser excitation. Infected erythrocytes, uninfected erythrocytes, and leukocytes were gated on logarithmic forward/side dot plots. Cells were analyzed at an average rate of 2000 to 3000 erythrocytes per second. Filters were placed before the green (FL-1) and red (FL-2) photomultiplier tubes (PMTs) such that the green PMT registered fluorescence emission between 520 and 555 nm, and the red PMT measured emission greater than 580 nm (*98*, *99*).

### Hemotoxicity assessment in humanized G6PD deficiency mouse model

Following procedures as previously published (*46*), hemotoxicity studies using huRBC engraftments were conducted in mice.

#### Animals

Female (8 to 9 weeks) NOD.CB17-Prkdcscid/J [nonobese diabetic severe combined immunodeficient (NOD/SCID)] mice were purchased from The Jackson Laboratory (Bar Harbor, ME). The animals were housed in a cage maintained in a specific pathogen–free animal facility and were acclimated for 1 week in the facility before initiation of experimental procedures. Sterile food and water were provided ad libitum throughout the study as previously described (*64*). All animal studies were performed under IACUC-approved protocols and adhere to the principles stated in the *Guide for the Care and Use of Laboratory Animals* (*100*).

#### Polymer therapeutic and TQ dosing

TQ was dissolved at a stock concentration of 1 mg/ml in 25% hydroxy-β-cyclodextrin acidified to pH 5 with acetic acid and diluted in DPBS for final dosing solutions. Polymer solutions for SC administration were prepared in DPBS at the final dosing concentrations. All drug concentrations were calculated per the free base molecular weights of TQ. The TQ parent compound was administered intragastrically via an intragastric feeder tube. Polymers and vehicle were administered SC in the right flank. TQ, pSVCTQ, and vehicle were given as a single dose on day 0. In this model, the TQ dose studied was 10 mg/kg; pSVCTQ was tested at doses of 2.5, 5.0, or 10 mg/kg TQ equivalent.

#### Hemotoxicity study design in the G6PD deficiency humanized mouse model

huRBCs from an A− G6PD–deficient donor (2.3 U/g Hgb) were transfused in NOD/SCID mice for 14 days as previously described (*64*). The percentage of huRBCs at the end of this period was analyzed by flow cytometry to evaluate glycophorin A (huRBCs) and Ter119 (mouse RBCs) circulating in peripheral blood. Only mice that had greater than 70% huRBC at the end of the transfusion period were used for subsequent assessment of hemolytic toxicity. The G6PD-deficient huRBC NOD/SCID mice were dosed with pSVCTQ or parent drug prepared as described above, with three to four mice per group. On days 4 and 7 following dosing, huRBC levels were assessed again by flow cytometry (glycophorin^+^Ter119^−^), along with the percentage of murine reticulocytes (CD71^+^Ter119) as an additional marker of hemolytic toxicity. Mice were euthanized on day 7 following initiation of treatment. Hematocrit and spleen weight were recorded as further markers of hemolytic toxicity (fig. S14).

### Anti-hypnozoite activity assessment

#### Ethics statement

The USAMD-AFRIMS IACUC Review Division, U.S. Army Medical Research and Materiel Command, reviewed and approved this study. Animals were maintained in accordance with established principles under the *Guide for the Care and Use of Laboratory Animals* (*100*) and the Animals for Scientific Purposes Act (*101*) and its subsequent regulations. The USAMD-AFRIMS animal care and use program is fully accredited by the AAALACi.

#### Cell culture and infection

Cryopreserved primary cynomolgus monkey hepatocytes (lot STX) and hepatocyte culture medium (HCM) (InVitroGro CP medium) were obtained from BioIVT Inc. (Baltimore, MD, USA) and thawed following the manufacturer’s recommendations. Hepatocytes were seeded in 384-well collagen-coated plates and used for experiments within 2 days after seeding as previously described (*52*). Infectious sporozoites were obtained from *Anopheles dirus* mosquitoes infected with *P. cynomolgi* B strain and were used to infect the plated primary NHP hepatocytes 2 days after seeding (*52*, *102*). HCM was changed the day after infection and every other day thereafter until drug treatment.

#### Drug treatment

pSVCTQ was dissolved in 1× PBS and used in either an 8-point or 12-point, twofold serial dilution at a final 100 or 500 μg/ml to 0.78 or 0.24 μg/ml concentrations. The treatment schedule was according to the radical cure mode where drug treatment starts 4 days after infection and continues for 4 days, with daily replacement of the old media with fresh drug-containing HCM (*52*, *102*–*105*).

#### Cell fixation and immunostaining

On day 8 after infection, the cells were washed with 1× PBS and incubated in 4% paraformaldehyde for 20 min at RT. Fixation was followed by three PBS washes and storage in 4°C before staining. For the visualization of *P. cynomolgi* parasites, a *Plasmodium*-specific glyceraldehyde-3-phosphate dehydrogenase (GAPDH) antibody (The European Malaria Reagent Repository) was added at 1.6 μg/ml in dilution buffer [1× PBS containing 1% (w/v) bovine serum albumin and 0.03% Triton X-100] and incubated at 4°C overnight. Subsequently, wells were washed thrice with 1× PBS and incubated at 4°C overnight with goat anti-mouse Alexa Fluor Plus 555 conjugate (2 μg/ml, catalog no. A32727, Invitrogen, Thermo Fisher Scientific, Waltham, MA, USA). Hoechst stain (10 μg/ml) was added to wells in dilution buffer, incubated for 1 hour at RT, then washed thrice, and filled with 1× PBS for imaging and storage. For ASGPR1 expression analysis, the mouse monoclonal anti-ASGPR1 (8D7) (2 μg/ml, catalog no. MA1-40244, Invitrogen, Thermo Fisher Scientific, Waltham, MA, USA) was added in dilution buffer and incubated at 4°C overnight. Afterward, the wells were washed thrice with 1× PBS and incubated at 4°C overnight with goat anti-mouse Alexa Fluor Plus 647 conjugate (2 μg/ml, catalog no. A32728TR, Invitrogen, Thermo Fisher Scientific, Waltham, MA, USA). Hoechst stain (10 μg/ml) was added to wells in dilution buffer, incubated for 1 hour at RT, then washed thrice, and filled with 1× PBS for imaging and storage.

#### Imaging and parasite detection

Imaging and image analysis of assay plates were completed using an Operetta CLS high-content imaging system and Harmony 4.9 software (PerkinElmer, Waltham, MA, USA). Images were acquired using tetramethyl rhodamine isocyanate (TRITC), DAPI, and bright-field channels at ×10 magnification. Using similar methodology described previously, parasites were counted with the TRITC channel and were identified by area, mean intensity, maximum intensity, and cell roundness parasites (*50*, *99**–**102*). Hepatocyte counts are generated from object detection via Hoechst stain intensity threshold and declumping algorithm. A secondary antibody–only stain control is used to generate erroneous counts, based on debris or background, and the average is subtracted from each well’s totals. Percent inhibition (PI) and toxicity values were calculated relative to in-plate negative controls for parasites and hepatocytes, respectively, using GraphPad Prism software (GraphPad Software Inc., USA, Windows version 9.5.1) using the average of experimental and biological replicates.

$$\text{PI}=100-\left(\frac{\mathrm{xy}}{\mu_{\text{negative control}}}\right)$$

### Manufacturability and COGS analysis

For the manufacturability analysis, each process step was evaluated by examining the starting materials and solvents with respect to safety and environmental concerns (as indicated in published Safety Data Sheets). Process conditions were evaluated in terms of any unusual conditions required (e.g., high temperature or pressure, highly reactive or flammable components), as well as assessing whether any equipment not typically found in a multipurpose specialty chemical manufacturing facility was required. All processes were concluded to be quite manufacturable using conventional equipment. The COGS analysis began with the demonstrated early laboratory results, with reasonable extrapolations for typical yield improvements that could be expected with process development work. Process improvement areas and their impacts were described, and the impact was estimated. Raw materials were evaluated by extrapolating current pricing at small scale to the pricing expected at the volumes required at commercial scale, with some projections for new compounds based on similar conventional compounds. Processing costs were developed by establishing equipment train size requirements (based on expected process improvements, cycle times, and projected campaign durations) and applying cost factors that account for the use of a given facility over a given time period. These processing costs include contractor direct costs (including labor and laboratory testing), indirect (overhead) costs, and typical profit margin for that type of business.

### Data analysis

Microsoft Excel and GraphPad Prism were used throughout the PK analysis to process the data, calculate pharmacokinetic parameters, and yield statistical information. A linear regression model was produced using the least squares fitting method with 1/*x*^2^ scaling for the assay calibrators. The AUC was determined using the linear trapezoidal rule, extended through 1 and 14 days for TQ datasets. For the G6PD deficiency model, data analysis was performed using GraphPad Prism (GraphPad Software). Mann-Whitney or one-way analysis of variance (ANOVA) with Bonferroni correction at 95% confidence intervals was used to determine differences between groups as appropriate. An *F* test comparing the two dose-response curves was also run. For all other work, a combination of Microsoft Excel and GraphPad Prism was used.

## Supplementary Materials

### This PDF file includes:

Supplementary Text Figs. S1 to S15 References
