# Supplementary material for: Liver-targeted polymeric prodrugs delivered subcutaneously improve tafenoquine therapeutic window for malaria radical cure
Source: Sci Adv. 2024 Apr 19;10(16):eadk4492. doi: 10.1126/sciadv.adk4492 (PMC11029812; doi:10.1126/sciadv.adk4492)
Supplement: Supplementary file 1 — Supplementary Text Figs. S1 to S15 References [file sciadv.adk4492_sm.pdf]

Science Advances
AAAS

Supplementary Materials for

Liver-targeted polymeric prodrugs delivered subcutaneously improve tafenoquine therapeutic window for malaria radical cure

Ayumi E. Pottenger *et al.*

Corresponding author: Patrick S. Stayton, stayton@uw.edu

Sci. Adv. **10**, eadk4492 (2024)
DOI: 10.1126/sciadv.adk4492

This PDF file includes:

Supplementary Text
Figs. S1 to S15
References

Supplementary Materials

Supplementary Text

Synthetic route for SVCTQ monomer

Synthesis of SMA-Val-Cit (VC) linker segment:
16mL of diethylamine (DEA) was added to Fmoc-Val-Cit (81, 106) (2.98 g, 6.0 mmol) in 64 mL DCM/ethanol (2:1) mixture and stirred at RT for 3 h. The solvent was rotary evaporated. 50 mL DCM was added to the residue and rotary evaporated (2 times). The residue was dissolved in 20% methanol/DCM (20 mL), precipitated into diethyl ether using four 50 mL conical centrifuge tubes (40 mL diethyl ether/tube), vortexed, and centrifuged. The pellets were transferred to a 250 mL conical flask, treated with 120 mL diethyl ether, and sonicated for 30 min. The product was filtered, rinsed with diethyl ether (30 mL) and dried under high vacuum for 24 h. Finally, the product was dissolved in 25 mL water and freeze-dried under vacuum for 3 days. Yield = 1.59 g (96.6 %).

Synthesis of stable SMA-Val-Cit-TQ Methacrylate (SVCTQMA):
A mixture of SMA-Val-Cit 487 mg (1 mmol), Tafenoquine (TQ) 509 mg (1.1 mmol) and *N*-ethoxycarbonyl-2-ethoxy-1,2-dihydroquinoline (EEDQ) 494 mg (2 mmol) in anhydrous dichloromethane (12 mL) and anhydrous methanol (3 mL) was stirred at RT. After 21 h, the reaction mixture was precipitated into 50% diethyl ether/pentane using four 50 mL conical centrifuge tubes (40 mL solvent mixture/tube), vortexed, and centrifuged to isolate the product. The pellet was washed with 50% diethyl ether/pentane (40 mL) by vortexing and centrifuging (2 times). The crude was purified by silica gel column chromatography using 8% methanol/chloroform. The column purified product in 7 mL 30 % methanol/chloroform was precipitated into 40 mL of 50% diethyl ether/pentane using one 50 mL conical centrifuge tube, vortexed and centrifuged. The pellet was washed with 50% diethyl ether/pentane (42 mL) by vortexing and centrifuging. The product was dried under high vacuum for 24 h to get SMA-Val-Cit-TQ (SVCTQMA). Yield = 416 mg (44.6 %).

Synthesis of p(GalNAcMA-co-SVCTQMA)

The RAFT copolymerization of GalNAc methacrylate (GalNAcMA) and SVCTQ methacrylate monomers was conducted in DMSO-d_6 under a nitrogen atmosphere using ECT as the CTA and V70 as the radical initiator. The initial monomer/CTA ratio ($[M]_0/[CTA]_0$) is 39:1 with a CTA/initiator ratio ($[CTA]_0/[I]_0$) of 1:0.14. The target mol% of GalNAc and SVCTQ was 87.5% and 12.5 % respectively. The target TQ drug wt% was 14.2 wt%. To a 10 mL round-bottomed flask equipped with a magnetic stir bar, was added DMSO-d_6 (total 3.42 mL), SVCTQ (200 mg, 0.2146 mmol), GalNAc (500 mg, 1.502 mmol), ECT (11.59 mg, 0.044 mmol), and V70 (1.90 mg, 0.0061 mmol). This mixture was vortexed for several minutes to give a homogeneous solution. A T_0 sample (20 μL) of the homogeneous solution was taken and stored at -20 °C for the determination of monomer conversion. The flask was then sealed with a rubber septum and the solution was degassed by bubbling nitrogen into the solution for 35 min. The flask was then placed in a pre-heated oil bath at 42 °C.

After 22 h, the reaction was stopped by introducing oxygen by removing the septa and cooling the solution with a mild stream of air. After the solution was cooled to ambient temperature, a T_f sample (20 μL) was taken and stored at -20 ° C for the determination of monomer conversion. The overall monomer conversion was obtained approximately 84%. The polymer was purified by precipitation, dialysis against DMSO and water. The lyophilized polymer was further purified by PD10 desalting column. About 488 mg powder polymer was obtained. The ^{1}H NMR spectroscopy demonstrated that the polymer was free from monomer impurities and the key proton resonance characteristics of SVCTQ and GalNAc were observed (Fig. S7).

The theoretical molecular weight was calculated to be 13,700 g/mol based on the targeted DP and monomer conversion. Absolute molecular weight of the polymer was 18 kDa based on 100% mass recovery. A relatively unimodal GPC trace was observed though the polymer has a complex macromolecular architecture (Fig. S9). The approximate DP of GalNAc and SVCTQ were approximately 29 and 4 respectively based on the targeted DP and the obtained monomer conversion. The TQ drug wt% was about 15 wt% calculated by ^{1}H NMR spectroscopy using levofloxacin as an internal standard. The characteristic proton of levofloxacin internal standard at 8.96 ppm (Fig. S8, **1, levofloxacin**) was compared with SVCTQ proton at 6.65 (Fig. S8, **2, SVCTQ**) ppm to calculate the TQ drug wt% (Fig. S8).

<u>Synthesis of p(GalNAcMA-*co*-SVCTQMA-*co*-RheMA)</u>

Rhodamine-labeled SVCTQ polymer was synthesized using rhodamine methacrylate (RheMA) as a comonomer with GalNAc and SVCTQ monomers. The synthetic process was similar to non-rhodamine labeled polymer. Briefly, the RAFT copolymerization of GAlNAc methacrylate, SVCTQ methacrylate, and rhodamine methacrylate monomers was conducted in DMSO-d_6 under a nitrogen atmosphere using ECT as the CTA and V70 as the radical initiator. The initial monomer/CTA ratio ($[M]_0/[CTA]_0$) is 39:1 with a CTA/initiator ratio ($[CTA]_0/[I]_0$) of 1:0.2. To a 5 mL round-bottomed flask equipped with a magnetic stir bar, was added DMSO-d_6 (total 2 mL), SVCTQ (120 mg, 0.1287 mmol), GalNAc (283.3 mg, 0.8499 mmol), RheMA (16 mg, 0.024 mmol), ECT (6.79 mg, 0.0258 mmol), and V70 (1.59 mg, 0.0051 mmol). The solution was degassed by bubbling nitrogen into the solution for 35 min. The flask was then placed in a pre-heated oil bath at 42 ° C for 22 h. The overall monomer conversion obtained was approximately 90%. The polymer was purified by precipitation, extensive dialysis against DMSO (until the solvent was colorless due to rhodamine) and cold water. The lyophilized polymer was further purified by PD10 desalting column. The ^{1}H NMR spectroscopy demonstrated that the polymer was free from any monomer impurities and the key proton resonance characteristics of SVCTQ, rhodamine, and GalNAc were observed (Fig. S10). The polymer turned red due to the incorporation of rhodamine. The theoretical molecular weight was calculated to be 14,700 g/mol based on the targeted DP and monomer conversion. The approximate DP of GalNAc, SVCTQ, and rhodamine were 30, 4 and 1 respectively based on the targeted DP and the obtained monomer conversion. The TQ drug wt% was about 15 wt% calculated by ^{1}H NMR spectroscopy using levofloxacin as the internal standard as illustrated previously (Fig. S8).

Supplementary Figures.

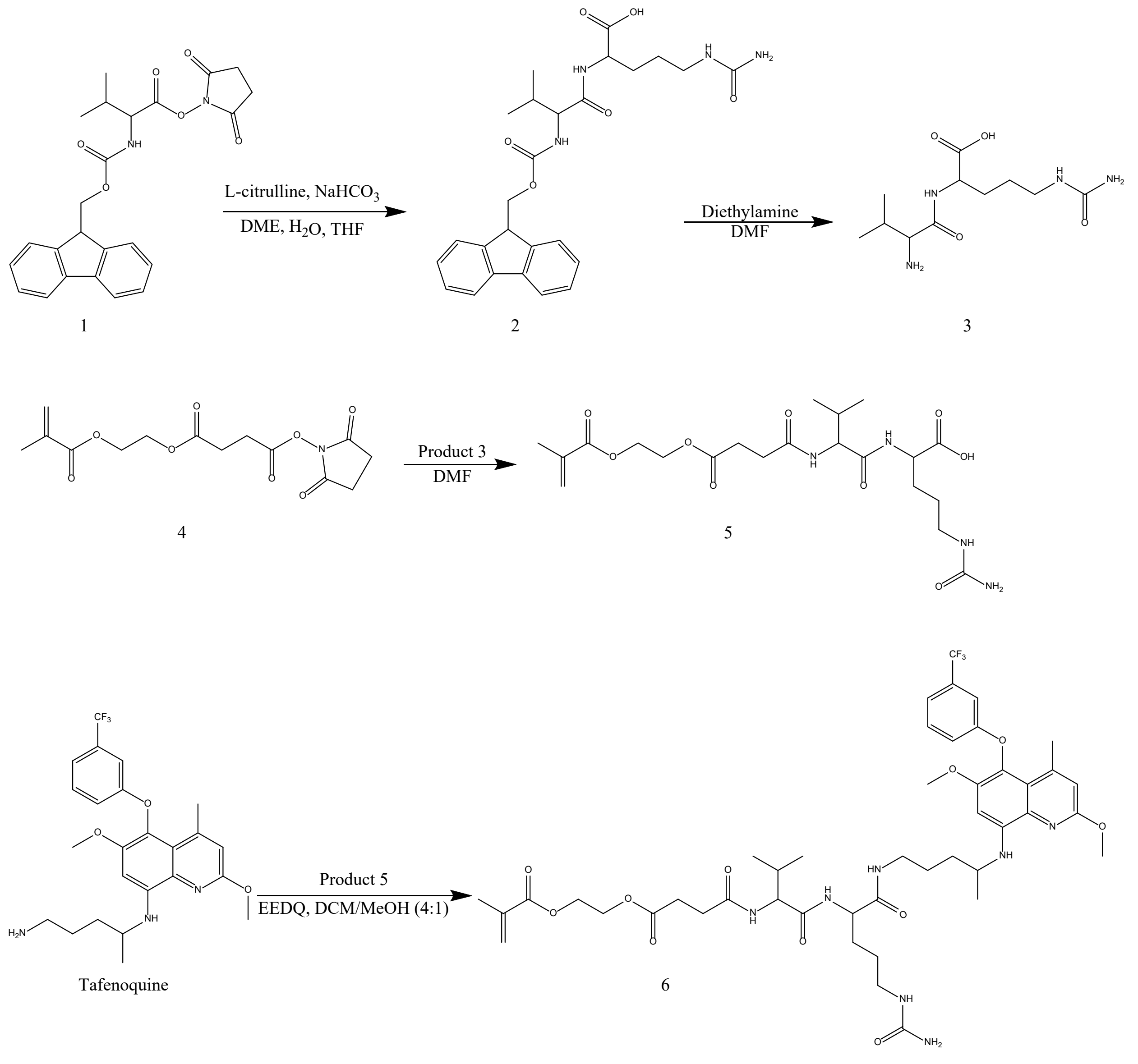

Fig. S1. Schematic of SVCTQ monomer synthesis.

Synthesis of enzyme-cleavable TQ prodrug monomer SVCTQ without a self-immolative p-aminobenzyl carbamate (PABC) spacer. Cathepsin B sensitive dipeptide linker Val-Cit (VC) was incorporated between the drug tafenoquine and the polymerizable methacrylate monomer.

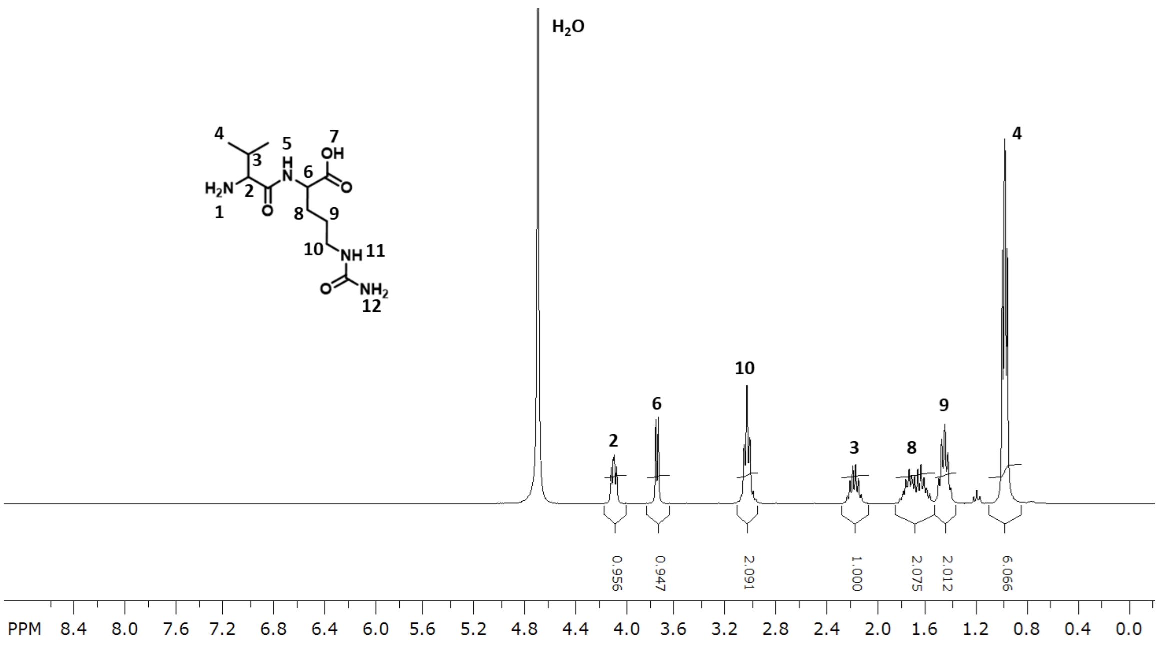

Fig. S2. ^{1}H-NMR spectrum of Val-Cit in D_2O.

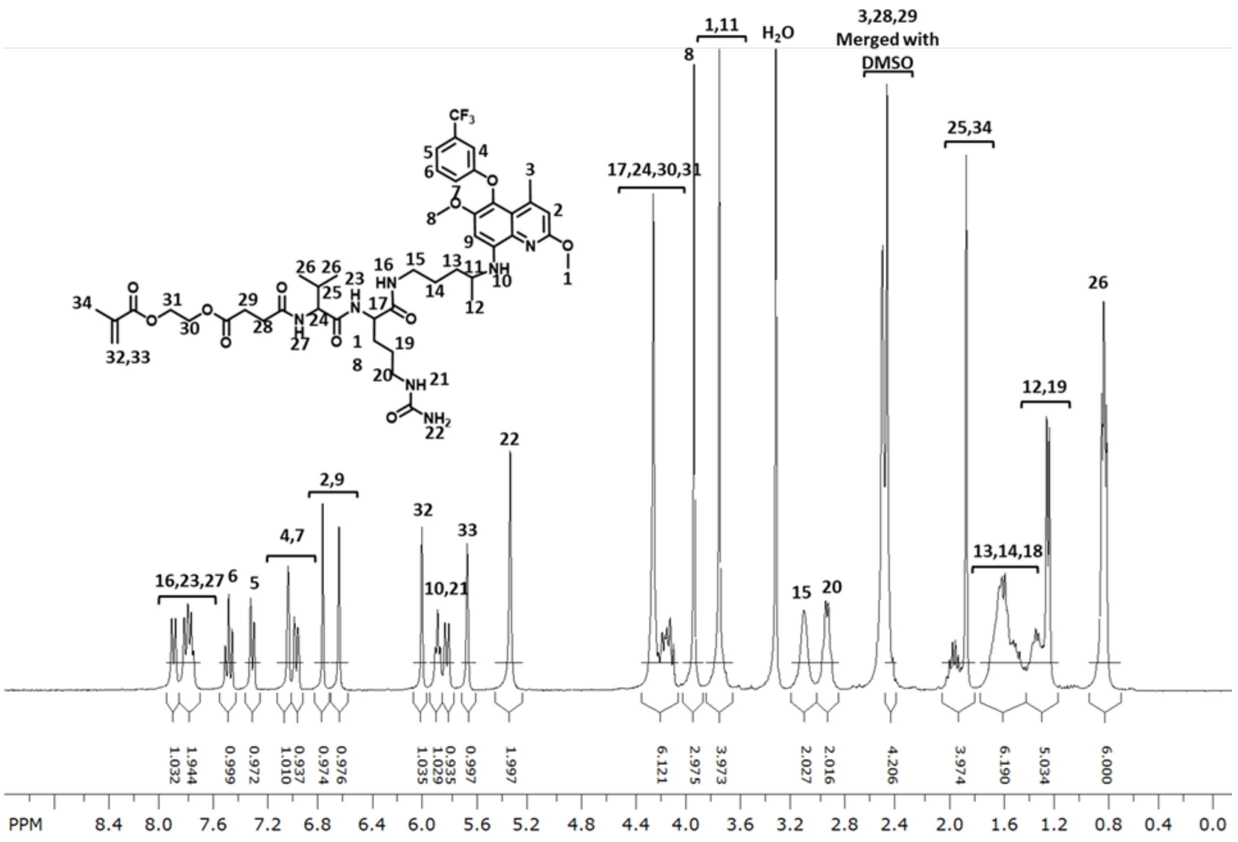

Fig. S3. ^{1}H-NMR spectrum of SMA-Val-Cit-TQ (SVCTQMA) in DMSO-d_6.

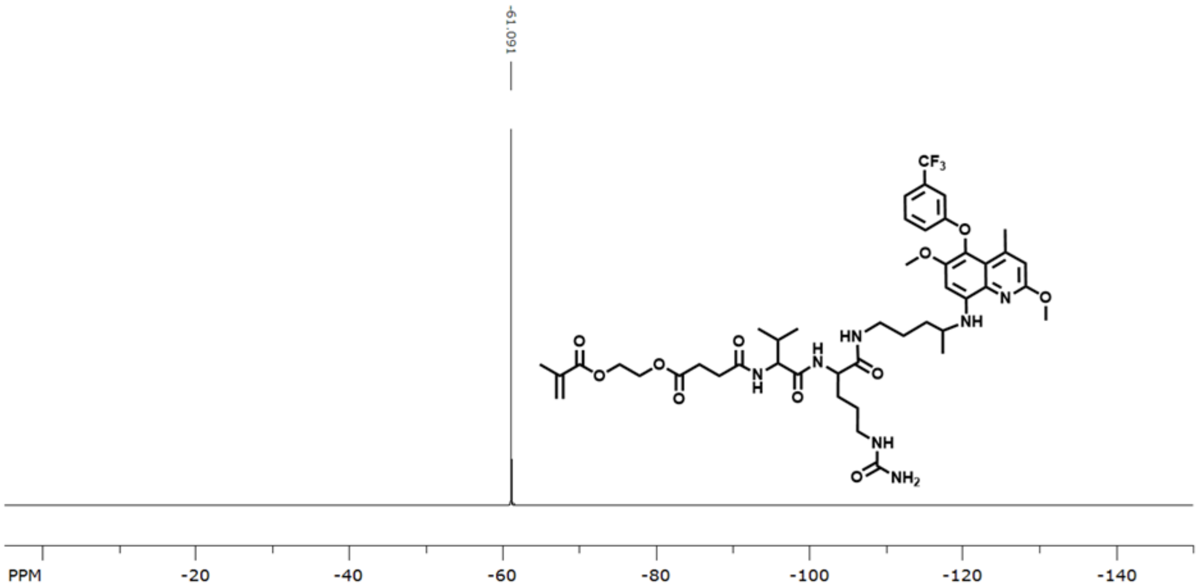

Fig. S4. ^{19}F-NMR spectrum of SMA-Val-Cit-TQ (SVCTQMA) in DMSO-d_6.

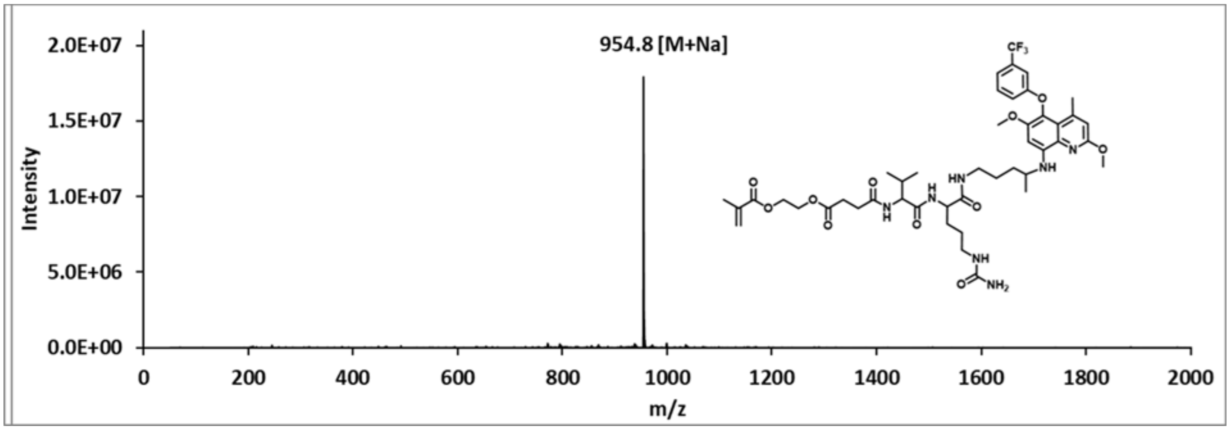

Fig. S5. ESI-Mass spectrum of SMA-Val-Cit-TQ (SVCTQMA).

Synthetic route for GalNAc-targeted SVCTQ polymers.

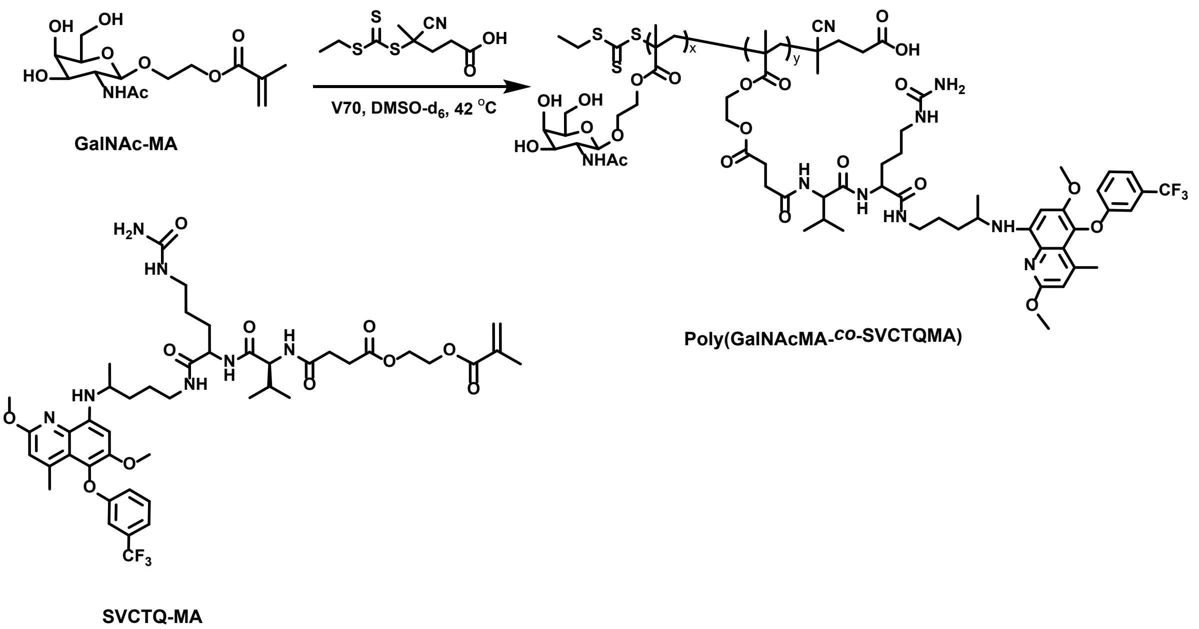

Fig. S6. Synthetic scheme for SVCTQ polymer (p(GalNAcMA-co-SVCTQMA)).

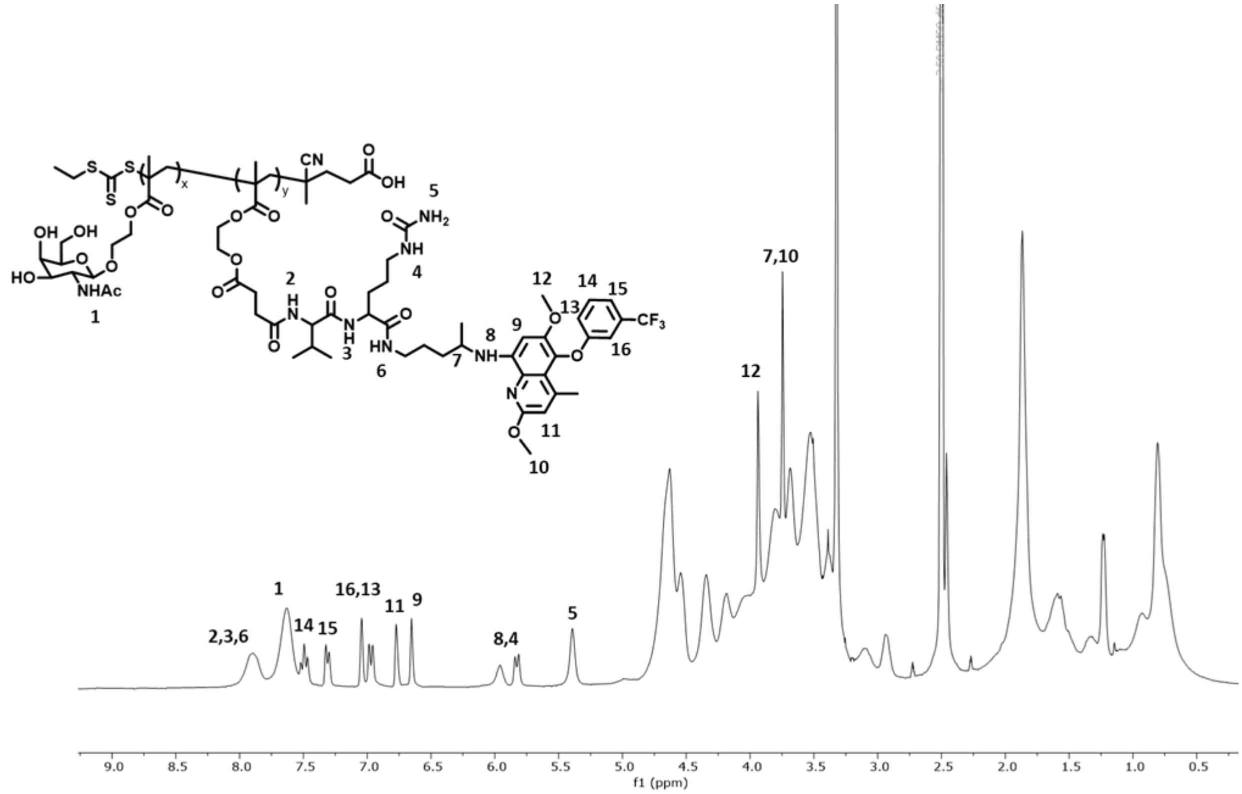

Fig. S7. ^{1}H NMR of p(GalNAcMA-co-SVCTQMA) in DMSO-d_6.

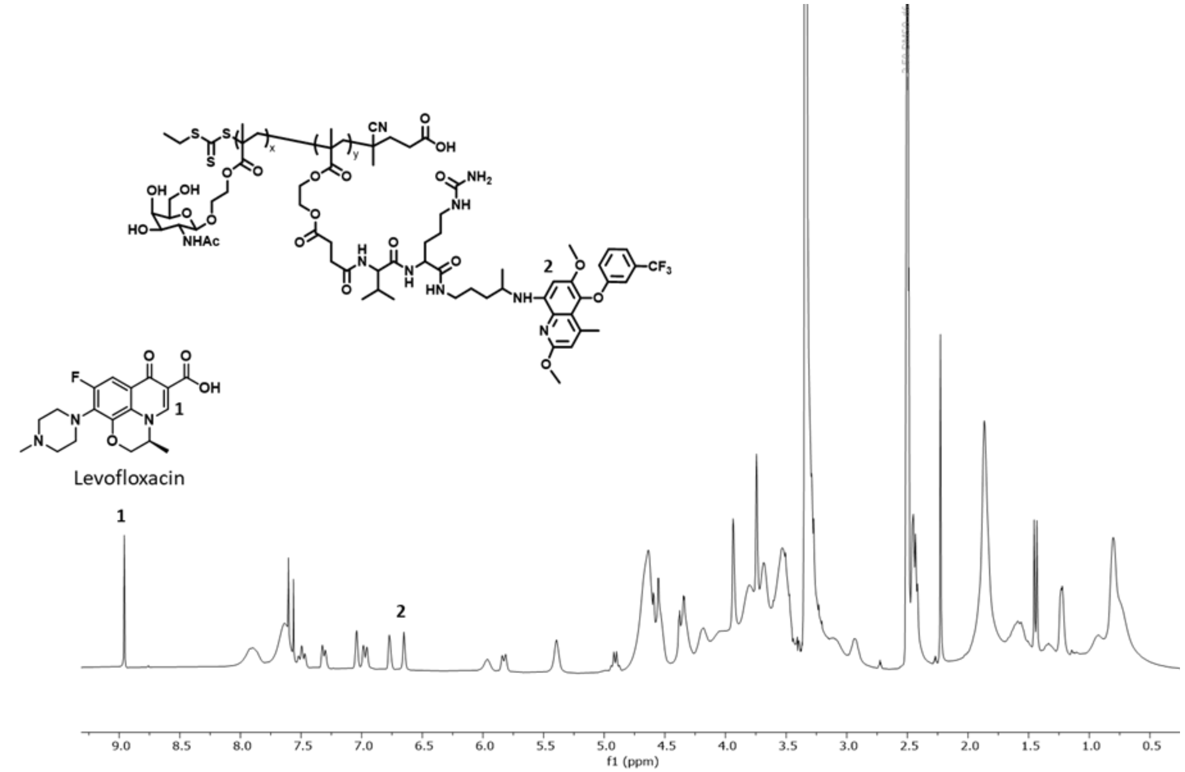

Fig. S8. ^{1}H NMR of p(GalNAcMA-co-SVCTQMA) in DMSO-d_6 using levofloxacin as an internal standard.

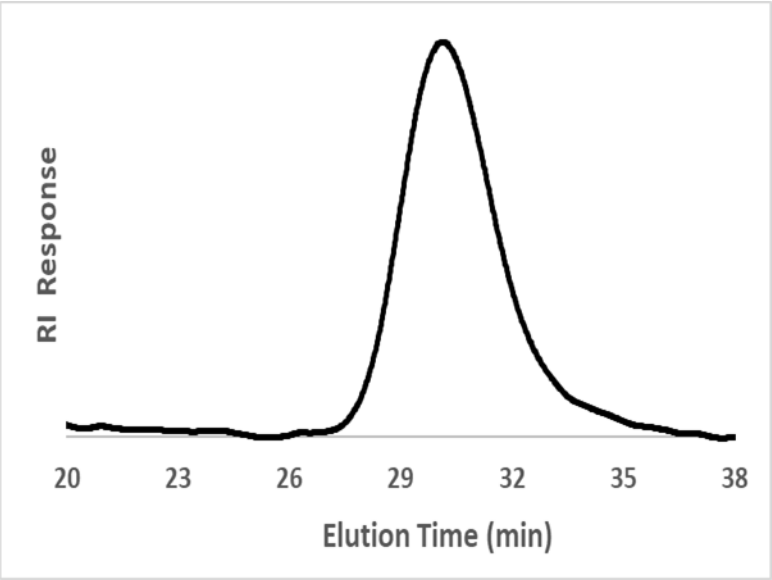

Fig. S9. Representative GPC trace of p(GalNAcMA-co-SVCTQMA) in LiBr-supplemented (0.1% w/v) DMF mobile phase at a flow rate of 1 mLmin^{-1}.

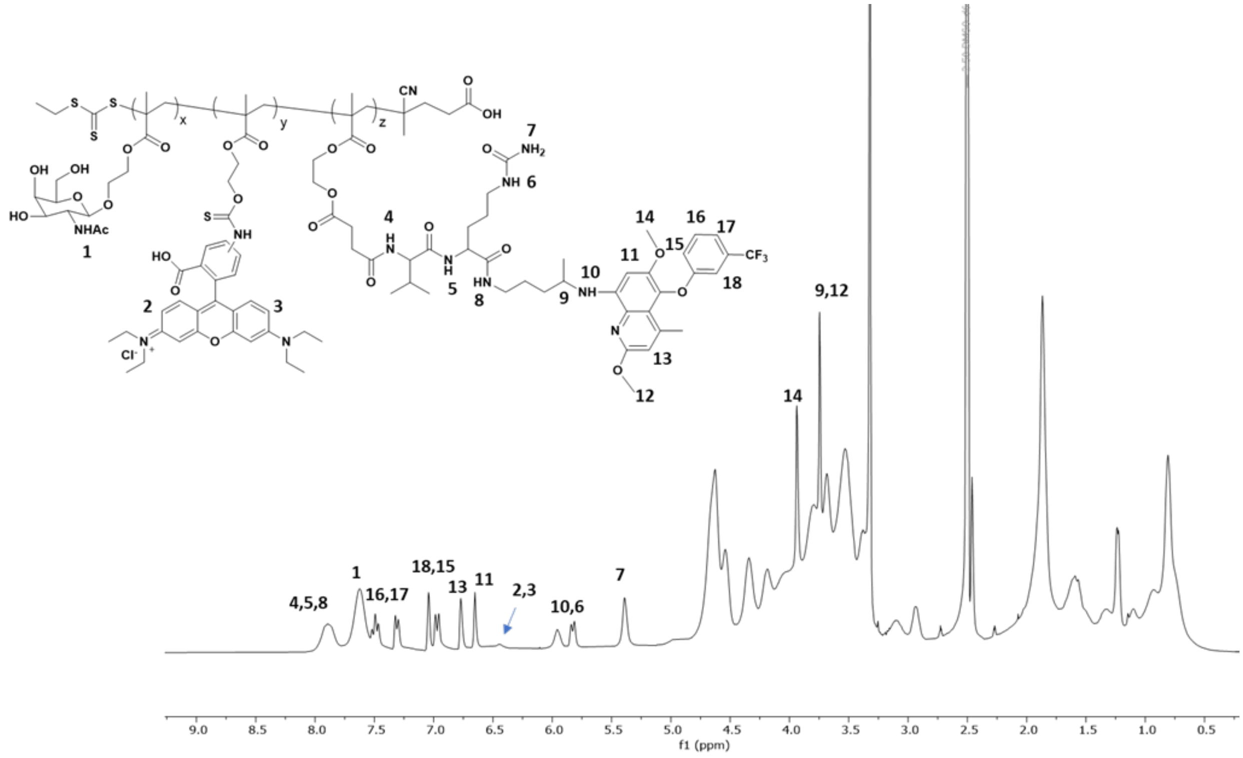

Fig. S10. ^{1}H NMR of p(GalNAcMA-co-SVCTQMA-co-RheMA) in DMSO-d_6.

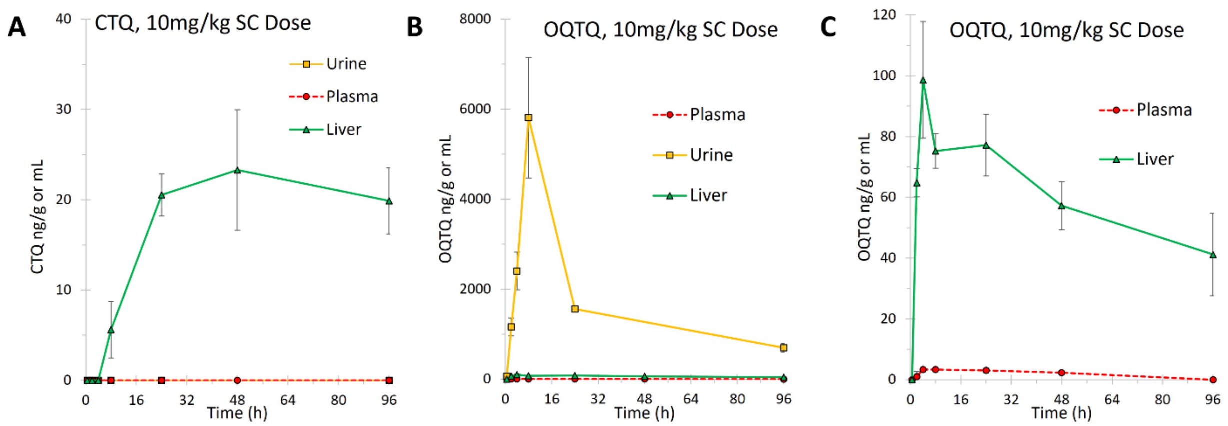

Fig. S11. PK of CTQ and OQTQ metabolites in liver, plasma, and urine.

Mouse pharmacokinetics of carboxy-TQ (CTQ) and 5,6 orthoquinone-TQ (OQTQ) after SC administration of the SVCTQ polymer. Time-course metabolite concentrations in liver, plasma, and urine were determined using LC-MS/MS. Each value represents the mean ± standard deviation (n = 3–4). (**A**) Liver, plasma, and urine concentration of CTQ after SC administration of a 10 mg/kg dose of SVCTQ polymer. (**B**) Liver, plasma, and urine concentration of OQTQ after SC administration of a 10 mg/kg dose of SVCTQ polymer. Not all timepoints had urine samples collected. See supplementary data. (**C**) Zoomed in to show plasma and liver concentration of OQTQ after SC administration of a 10 mg/kg dose of SVCTQ polymer. Y axis: Liver is in ng/g liver; Plasma is in ng/mL plasma; Urine is in ng/mL urine. Final figure made with Biorender.com.

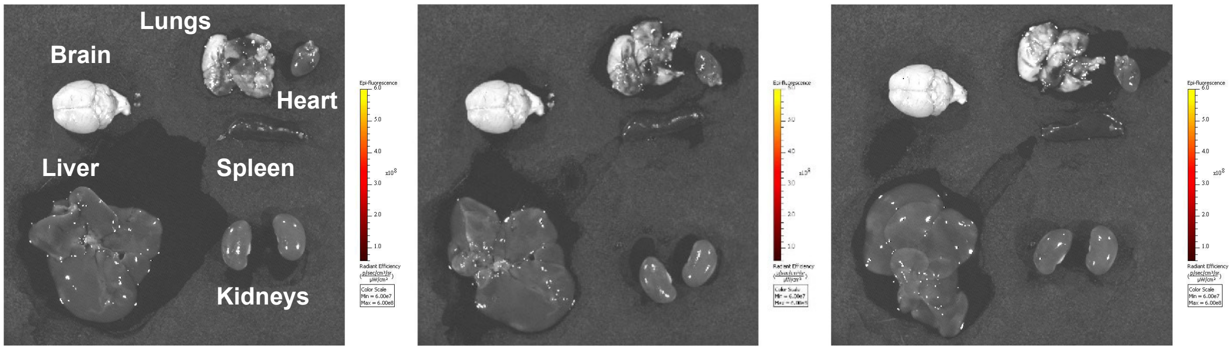

Fig. S12. IVIS biodistribution controls.

IVIS imaging of relevant tissues at 8h post-SC injection of vehicle control (PBS) in mice (n=3) using Xenogen Living Image software. Images show what level of background fluorescence is to be expected in these tissues.

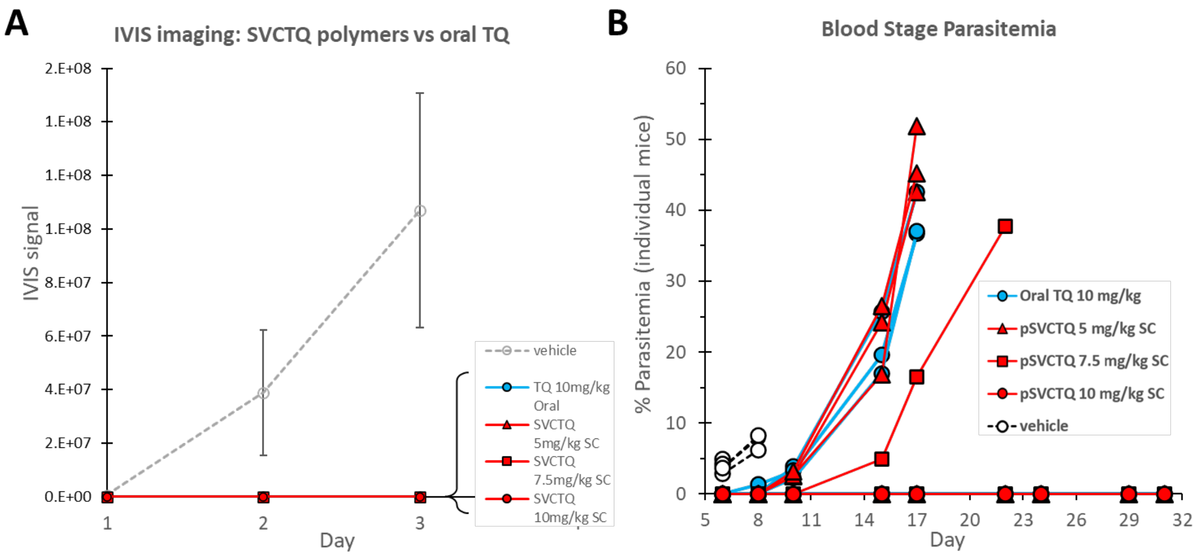

Fig. S13. Efficacy of SVCTQ polymer and oral TQ in *P. berghei* causal prophylaxis model during liver-stage of infection.

Mice were administered a single dose of either TQ (10 mg/kg, PO) or SVCTQ polymer (5, 7.5, or 10 mg/kg, SC), or vehicle (DPBS, SC) on Day −1 with respect to sporozoite infection (n = 5) (**A**) IVIS imaging on Days 1 through 3. The IVIS signal measured at each timepoint is correlated with the levels of luciferase-expressing *P. berghei* in the liver. IVIS signal, measured in total flux of photons, measured at each timepoint is correlated with the levels of luciferase-expressing *P. berghei in* the liver. Lack of IVIS signal indicates a suppression of sporozoite proliferation in the liver. (**B**) Blood stage parasitemia results over 31 days for each individual mouse. 0/5 vehicle-treated mice survived; 2/5 oral TQ-treated mice survived; 2/5 5mg/kg polymer-treated mice survived; 4/5 7.5mg/kg polymer-treated mice survived; 5/5 10mg/kg polymer-treated mice survived.

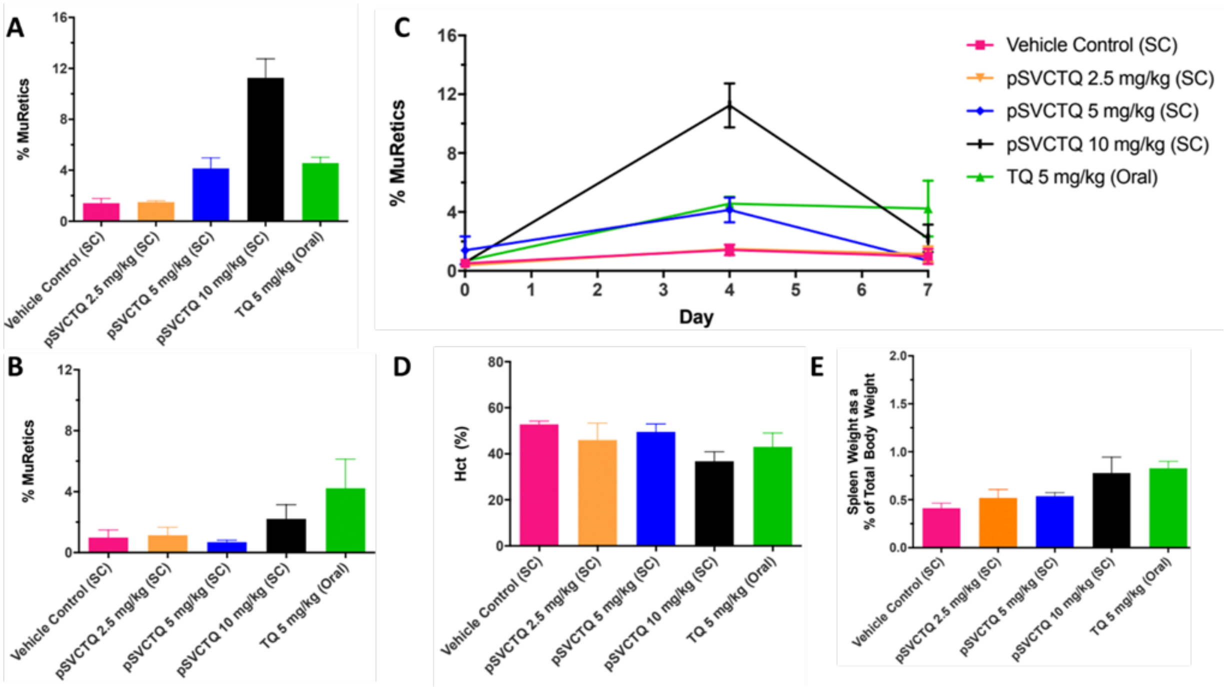

Fig. S14. Reticulocyte, hematocrit, and spleen weight following SVCTQ polymer administration in a mouse hemotoxicity model.

(**A**) Peripheral blood levels of mouse reticulocytes on Day 4 (**B**) Peripheral blood levels of mouse reticulocytes on Day 7. (**C**) Kinetics of mouse reticulocyte production. (**D**) Percent hematocrit (HCT) levels on Day 7. (**E**) Spleen weight on Day 7. SC: Subcutaneous; pSVCTQ: SVCTQ polymer; TQ: tafenoquine.

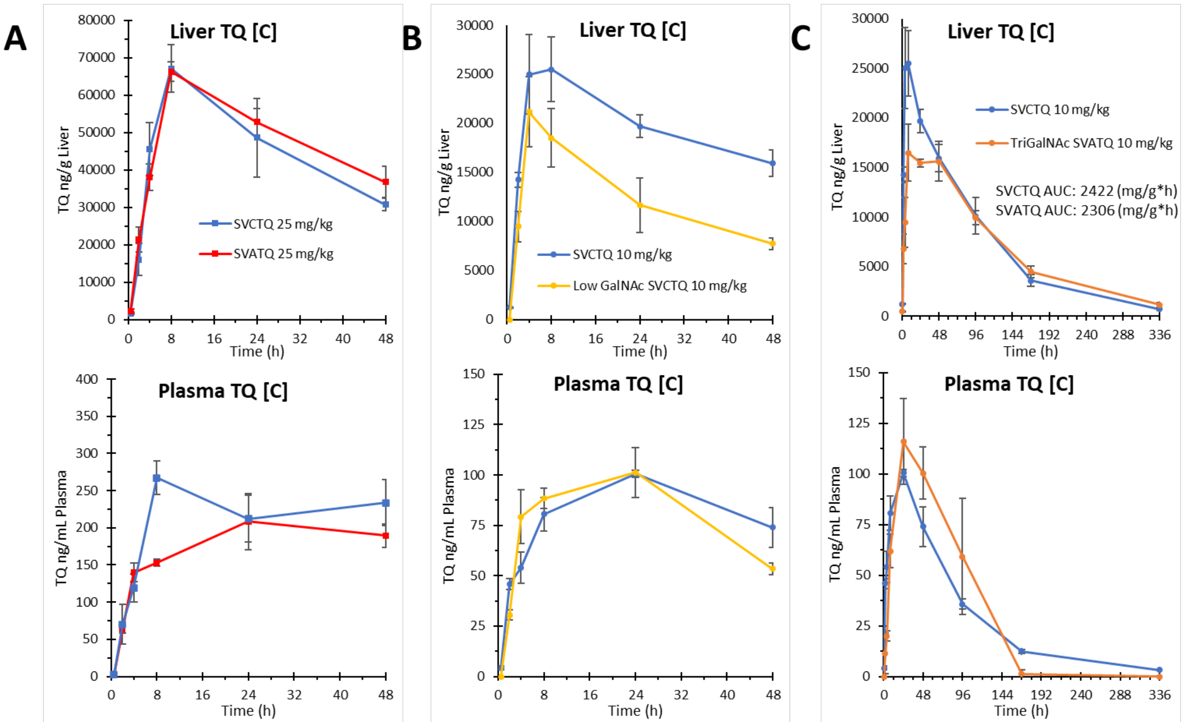

Fig. S15. PK of TQ in liver after SVCTQ, SVATQ or tri-antennary GalNAc polymer administration.

Mouse pharmacokinetics (PK) of tafenoquine (TQ) after SC administration of the p(GalNAc-co-SVCTQMA) (SVCTQ), p(GalNAc-co-SVATQMA) (SVATQ), p(GalNAc-co-MSEMA-co-SVCTQMA) (Low GalNAc SVCTQMA), and the p(GMA-co-SVATQMA) (TriGalNAc SVATQMA) polymers (where GMA = Glycerol monomethacrylate, tri-antennary GalNAc CTA used to RAFT synthesize polymer) . Time-course TQ concentrations in liver were determined using LC-MS/MS. Each value represents the mean ± standard deviation (n = 3). (**A**) TQ concentration in liver and plasma after SC administration of a 25 mg/kg dose of SVCTQ or SVATQ polymer. (**B**) TQ concentration in liver and plasma after SC administration of a 10 mg/kg dose of SVCTQ or Low GalNAc SVCTQ polymer. (**C**) TQ concentration in liver and plasma after SC administration of a 10 mg/kg dose of TriGalNAc SVATQ polymer. Y axis: Liver is ng/g liver, plasma is in ng/mL plasma.

REFERENCES AND NOTES

1. C. Kimeu, “Eliminate malaria once and for all or it will come back stronger, UN warned,” The Guardian, 22 September 2023; https://theguardian.com/global development/2023/sep/22/eliminate-malaria-once-and-for-all-or-it-will-come-back-stronger-un-warned.

2. Global Malaria Programme, World Health Organization, World Malaria Report 2022; https://who.int/teams/global-malaria-programme/reports/world-malaria-report-2022.

3. K. E. Battle, T. C. D. Lucas, M. Nguyen, R. E. Howes, A. K. Nandi, K. A. Twohig, D.A. Pfeffer, E. Cameron, P. C. Rao, D. Casey, H. S. Gibson, J. A. Rozier, U. Dalrymple, S. H. Keddie, E. L. Collins, J. R. Harris, C. A. Guerra, M. P. Thorn, D. Bisanzio, N. Fullman, C. K. Huynh, X. Kulikoff, M. J. Kutz, A. D. Lopez, A. H. Mokdad, M. Naghavi, G. Nguyen, K. A. Shackelford, T. Vos, H. Wang, S. S. Lim, C. J. L. Murray, R. N. Price, J. K. Baird, D. L. Smith, S. Bhatt, D. J. Weiss, S. I. Hay, P. W. Gething, Mapping the global endemicity and clinical burden of Plasmodium vivax, 2000-17: A spatial and temporal modelling study. Lancet 394, 332–343 (2019).

4. S. Hundessa, G. Williams, S. Li, L. Liu, W. Cao, H. Ren, J. Guo, A. Gasparrini, K. Ebi, W. Zhang, Y. Guo, Projecting potential spatial and temporal changes in the distribution of Plasmodium vivax and Plasmodium falciparum malaria in China with climate change. Sci. Total Environ. 627, 1285–1293 (2018).

5. A. J. Trajer, The changing risk patterns of Plasmodium vivax malaria in Greece due to climate change. Int. J. Environ. Health Res. 32, 665–690 (2022).

6. R. N. Price, N. M. Douglas, Expanding the use of primaquine for the radical cure of Plasmodium vivax. Clin. Infect. Dis. 67, 1008–1009 (2018).

7. K. Mendis, B. J. Sina, P. Marchesini, R. Carter, The neglected burden of Plasmodium vivax malaria. Am. J. Trop. Med. Hyg. 64, 97–106 (2001).

8. S. D. Fernando, D. M. Gunawardena, M. R. Bandara, D. De Silva, R. Carter, K. N. Mendis, A. R. Wickremasinghe, The impact of repeated malaria attacks on the school performance of children. Am. J. Trop. Med. Hyg. 69, 582–588 (2003).

9. K. A. Moore, J. A. Simpson, M. J. L. Scoullar, R. McGready, F. J. I. Fowkes, Quantification of the association between malaria in pregnancy and stillbirth: A systematic review and meta-analysis. Lancet Glob. Health 5, e1101–e1112 (2017).

10. A. P. Phyo, P. Dahal, M. Mayxay, E. A. Ashley, Clinical impact of vivax malaria: A collection review. PLOS Med. 19, e1003890 (2022).

11. R. Tapajos, D. Castro, G. Melo, S. Balogun, M. James, R. Pessoa, A. Almeida, M. Costa, R. Pinto, B. Albuquerque, W. Monteiro, J. Braga, M. Lacerda, M. P. Mourao, Malaria impact on cognitive function of children in a peri-urban community in the Brazilian Amazon. Malar. J. 18, 173 (2019).

12. J. R. Poespoprodjo, W. Fobia, E. Kenangalem, D. A. Lampah, A. Hasanuddin, N. Warikar, P. Sugiarto, E. Tjitra, N. M. Anstey, R. N. Price, Vivax malaria: A major cause of morbidity in early infancy. Clin. Infect. Dis. 48, 1704–1712 (2009).

13. N. J. White, Determinants of relapse periodicity in Plasmodium vivax malaria. Malar. J. 10, 297 (2011).

14. J. C. Haston, J. Hwang, K. R. Tan, Guidance for using tafenoquine for prevention and antirelapse therapy for malaria—United States, 2019. MMWR Morb. Mortal. Wkly Rep. 68, 1062–1068 (2019).

15. ARAKODA (tafenoquine) tablets. Highlights of Prescribing Information (United States Food and Drug Administration, 2018); https://accessdata.fda.gov/drugsatfda_docs/label/2018/210607lbl.pdf.

16. A. Mayence, J. J. Vanden Eynde, Tafenoquine: A 2018 novel FDA-approved prodrug for the radical cure of Plasmodium vivax malaria and prophylaxis of malaria. Pharmaceuticals 12, 115–121 (2019).

17. KRINTAFEL (tafenoquine) tablets. Highlights of Prescribing Information (United States Food and Drug Administration, 2018); https://accessdata.fda.gov/drugsatfda_docs/label/2018/210795s000lbl.pdf.

18. J. A. Green, K. Mohamed, N. Goyal, S. Bouhired, A. Hussaini, S. W. Jones, G. Koh, I. Kostov, M. Taylor, A. Wolstenholm, S. Duparc, Pharmacokinetic interactions between tafenoquine and dihydroartemisinin-piperaquine or artemether-lumefantrine in healthy adult subjects. Antimicrob. Agents Chemother. 60, 7321–7332 (2016).

19. M. V. G. Lacerda, A. Llanos-Cuentas, S. Krudsood, C. Lon, D. L. Saunders, R. Mohammed, D. Yilma, D. B. Pereira, F. E. J. Espino, R. Z. Mia, R. Chuquiyauri, F. Val, M. Casapia, W. M. Monteiro, M. A. M. Brito, M. R. F. Costa, N. Buathong, H. Noedl, E. Diro, S. Getie, K. M. Wubie, A. Abdissa, A. Zeynudin, C. Abebe, M. S. Tada, F. Brand, H. P. Beck, B. Angus, S. Duparc, J. P. Kleim, L. M. Kellam, V. M. Rousell, S. W. Jones, E. Hardaker, K. Mohamed, D. D. Clover, K. Fletcher, J. J. Breton, C. O. Ugwuegbulam, J. A. Green, G. Koh, Single-dose tafenoquine to prevent relapse of Plasmodium vivax malaria. N. Engl. J. Med. 380, 215–228 (2019).

20. N. M. Douglas, J. R. Poespoprodjo, D. Patriani, M. J. Malloy, E. Kenangalem, P. Sugiarto, J. A. Simpson, Y. Soenarto, N. M. Anstey, R. N. Price, Unsupervised primaquine for the treatment of Plasmodium vivax malaria relapses in southern Papua: A hospital-based cohort study. PLOS Med. 14, e1002379 (2017).

21. K. Thriemer, A. Bobogare, B. Ley, C. S. Gudo, M. S. Alam, N. M. Anstey, E. Ashley, J. K. Baird, C. Gryseels, E. Jambert, M. Lacerda, F. Laihad, J. Marfurt, A. P. Pasaribu, J. R. Poespoprodjo, I. Sutanto, W. R. Taylor, C. van den Boogaard, K. E. Battle, L. Dysoley, P. Ghimire, B. Hawley, J. Hwang, W. A. Khan, R. N. B. Mudin, M. E. Sumiwi, R. Ahmed, M. M. Aktaruzzaman, K. R. Awasthi, A. Bardaji, D. Bell, L. Boaz, F. H.

Burdam, D. Chandramohan, Q. Cheng, K. Chindawongsa, J. Culpepper, S. Das, R. Deray, M. Desai, G. Domingo, D. Q. Wang, S. Duparc, R. Floranita, E. Gerth-Guyette, R. E. Howes, C. Hugo, G. Jagoe, E. Sariwati, S. T. Jhora, J. W. Wu, H. Karunajeewa, E. Kenangalem, B. K. Lal, C. Landuwulang, E. Le Perru, S. E. Lee, L. S. Makita, J. McCarthy, A. Mekuria, N. Mishra, E. Naket, S. Nambanya, J. Nausien, T. N. Duc, T. N. Thi, R. Noviyanti, D. Pfeffer, G. Qi, A. Rahmalia, S. Rogerson, I. Samad, J. Sattabongkot, A. Satyagraha, D. Shanks, S. N. Sharma, C. H. Sibley, A. Sungkar, D. Syafruddin, A. Talukdar, J. Tarning, F. ter Kuile, S. Thapa, M. Theodora, T. T. Huy, E. Waramin, G. Waramori, A. Woyessa, C. Wongsrichanalai, N. X. Xa, J. S. Yeom, L. Hermawan, A. Devine, S. Nowak, I. Jaya, S. Supargiyono, K. P. Grietens, R. N. Price, Quantifying primaquine effectiveness and improving adherence: A round table discussion of the APMEN Vivax Working Group. Malar. J. 17, 241 (2018).

22. R. Takeuchi, S. Lawpoolsri, M. Imwong, J. Kobayashi, J. Kaewkungwal, S. Pukrittayakamee, S. Puangsa-Art, N. Thanyavanich, W. Maneeboonyang, N. P. J. Day, P. Singhasivanon, Directly-observed therapy (DOT) for the radical 14-day primaquine treatment of Plasmodium vivax malaria on the Thai-Myanmar border. Malar. J. 9, 308 (2010).

23. E. T. Nkhoma, C. Poole, V. Vannappagari, S. A. Hall, E. Beutler, The global prevalence of glucose-6-phosphate dehydrogenase deficiency: A systematic review and metaanalysis. Blood Cells Mol. Dis. 42, 267–278 (2009).

24. J. Recht, E. Ashley, N. White, Safety of 8-aminoquinoline antimalarial medicines (World Health Organization & Mahidol Oxford Research Unit, 2014); https://apps.who.int/iris/handle/10665/112735.

25. R. E. Howes, F. B. Piel, A. P. Patil, O. A. Nyangiri, P. W. Gething, M. Dewi, M. M. Hogg, K. E. Battle, C. D. Padilla, J. K. Baird, S. I. Hay, G6PD deficiency prevalence and estimates of affected populations in malaria endemic countries: A geostatistical model-based map. PLOS Med. 9, e1001339 (2012).

26. B. Galatas, L. Mabote, W. Simone, G. Matambisso, L. Nhamussua, M. D. Manu-Pereira, C. Menendez, F. Saute, E. Macete, Q. Bassat, P. Alonso, P. Aide, Heterogeneity of G6PD deficiency prevalence in Mozambique: A school-based cross-sectional survey in three different regions. Malar. J. 16, 36 (2017).

27. E. A. Ashley, J. Recht, N. J. White, Primaquine: The risks and the benefits. Malar. J. 13, 418 (2014).

28. J. Recht, E. A. Ashley, N. J. White, Use of primaquine and glucose-6-phosphate dehydrogenase deficiency testing: Divergent policies and practices in malaria endemic countries. PLOS Negl. Trop. Dis. 12, e0006230 (2018).

29. N. J. White, The antimalarial activity of tafenoquine in falciparum malaria. Clin. Infect. Dis. 76, 1928–1929 (2023).

30. M. B. Markus, Safety and efficacy of tafenoquine for Plasmodium vivax malaria prophylaxis and radical cure: Overview and perspectives. Ther. Clin. Risk Manag. 17, 989–999 (2021).

31. P. Melariri, L. Kalombo, P. Nkuna, A. Dube, R. Hayeshi, B. Ogutu, L. Gibhard, C. deKock, P. Smith, L. Wiesner, H. Swai, Oral lipid-based nanoformulation of tafenoquine enhanced bioavailability and blood stage antimalarial efficacy and led to a

reduction in human red blood cell loss in mice. Int. J. Nanomedicine 10, 1493–1503 (2015).

32. L. N. Borgheti-Cardoso, S. A. A. Kooijmans, L. G. Chamorro, A. Biosca, E. Lantero, M. Ramirez, Y. Avalos-Padilla, I. Crespo, I. Fernandez, C. Fernandez-Becerra, H. A. Del Portillo, X. Fernandez-Busquets, Extracellular vesicles derived from Plasmodiuminfected and non-infected red blood cells as targeted drug delivery vehicles. Int. J. Pharm. 587, 119627 (2020).

33. E. Moles, M. Kavallaris, X. Fernandez-Busquets, Modeling the distribution of diprotic basic drugs in liposomal systems: Perspectives on malaria nanotherapy. Front. Pharmacol. 10, 1064 (2019).

34. L. Fortuin, M. Leshabane, R. Pfukwa, D. Coertzen, L.-M. Birkholtz, B. Klumperman, Facile route to targeted, biodegradable polymeric prodrugs for the delivery of combination therapy for malaria. ACS Biomater Sci. Eng. 6, 6217–6227 (2020).

35. S. Jokonya, M. Langlais, M. Leshabane, P. W. Reader, J. A. Vosloo, R. Pfukwa, D. Coertzen, L. M. Birkholtz, M. Rautenbach, B. Klumperman, Poly(N-vinylpyrrolidone) antimalaria conjugates of membrane-disruptive peptides. Biomacromolecules 21, 5053–5066 (2020).

36. R. Rueangweerayut, G. Bancone, E. J. Harrell, A. P. Beelen, S. Kongpatanakul, J. J. Mohrle, V. Rousell, K. Mohamed, A. Qureshi, S. Narayan, N. Yubon, A. Miller, F. H. Nosten, L. Luzzatto, S. Duparc, J. P. Kleim, J. A. Green, Hemolytic potential of tafenoquine in female volunteers heterozygous for glucose-6-phosphate dehydrogenase (G6PD) deficiency (G6PD mahidol variant) versus G6PD-normal volunteers. Am. J. Trop. Med. Hyg. 97, 702–711 (2017).

37. Southeast Asia Dose Optimization of Tafenoquine (SEADOT). ClinicalTrials.gov Identifier: NCT04704999 (2021); https://classic.clinicaltrials.gov/ct2/show/NCT0470499 9.

38. J. A. Watson, R. J. Commons, J. Tarning, J. A. Simpson, A. Llanos Cuentas, M. V. G. Lacerda, J. A. Green, G. C. K. W. Koh, C. S. Chu, F. H. Nosten, R. N. Price, N. P. J. Day, N. J. White, The clinical pharmacology of tafenoquine in the radical cure of Plasmodium vivax malaria: An individual patient data meta-analysis. eLife 11, e83433 (2022).

39. J. Kopeček, R. Duncan, Targetable polymeric prodrugs. J. Control. Release 6, 315–327 (1987).

40. J. L. Vennerstrom, E. O. Nuzum, R. E. Miller, A. Dorn, L. Gerena, P. A. Dande, W. Y. Ellis, R. G. Ridley, W. K. Milhous, 8-Aminoquinolines active against blood stage Plasmodium falciparum in vitro inhibit hematin polymerization. Antimicrob. Agents Chemother. 43, 598–602 (1999).

41. J. C. Anders, H. Chung, A. D. Theoharides, Methemoglobin formation resulting from administration of candidate 8-aminoquinoline antiparasitic drugs in the dog. Fundam. Appl. Toxicol. 10, 270–275 (1988).

42. Y. C. Lee, R. R. Townsend, M. R. Hardy, J. Lonngren, J. Arnarp, M. Haraldsson, H. Lonn, Binding of synthetic oligosaccharides to the hepatic Gal/GalNAc lectin. Dependence on fine structural features. J. Biol. Chem. 258, 199–202 (1983).

43. V. K. Sharma, M. F. Osborn, M. R. Hassler, D. Echeverria, S. Ly, E. A. Ulashchik, Y. V. Martynenko-Makaev, V. V. Shmanai, T. S. Zatsepin, A. Khvorova, J. K. Watts, Novel cluster and monomer-based GalNAc structures induce effective uptake of siRNAs in vitro and in vivo. Bioconjug. Chem. 29, 2478–2488 (2018).

44. P. D. Senter, E. L. Sievers, The discovery and development of brentuximab vedotin for use in relapsed Hodgkin lymphoma and systemic anaplastic large cell lymphoma. Nat. Biotechnol. 30, 631–637 (2012).

45. M. R. Gordon, M. Canakci, L. Li, J. Zhuang, B. Osborne, S. Thayumanavan, Field guide to challenges and opportunities in antibody-drug conjugates for chemists. Bioconjug. Chem. 26, 2198–2215 (2015).

46. S. Srinivasan, D. Roy, T. E. J. Chavas, V. Vlaskin, D. K. Ho, A. Pottenger, C. L. M. LeGuyader, M. Maktabi, P. Strauch, C. Jackson, S. M. Flaherty, H. Lin, J. Zhang, B. Pybus, Q. Li, H. E. Huber, P. A. Burke, D. Wesche, R. Rochford, P. S. Stayton, Livertargeted polymeric prodrugs of 8-aminoquinolines for malaria radical cure. J. Control. Release 331, 213–227 (2021).

47. M. Dorywalska, R. Dushin, L. Moine, S. E. Farias, D. Zhou, T. Navaratnam, V. Lui, A. Hasa-Moreno, M. G. Casas, T. T. Tran, K. Delaria, S. H. Liu, D. Foletti, C. J. O'Donnell, J. Pons, D. L. Shelton, A. Rajpal, P. Strop, Molecular basis of valinecitrulline-PABC linker instability in site-specific ADCs and its mitigation by linker design. Mol. Cancer Ther. 15, 958–970 (2016).

48. Y. Anami, C. M. Yamazaki, W. Xiong, X. Gui, N. Zhang, Z. An, K. Tsuchikama, Glutamic acid-valine-citrulline linkers ensure stability and efficacy of antibody-drug conjugates in mice. Nat. Commun. 9, 2512 (2018).

49. F. Macintyre, H. Ramachandruni, J. N. Burrows, R. Holm, A. Thomas, J. J. Mohrle, S.

Duparc, R. Hooft van Huijsduijnen, B. Greenwood, W. E. Gutteridge, T. N. C. Wells, W. Kaszubska, Injectable anti-malarials revisited: Discovery and development of new agents to protect against malaria. Malar. J. 17, 402 (2018).

50. L. L. Fonseca, C. J. Joyner, C. L. Saney, The MaHPIC Consortium, A. Moreno, J. W. Barnwell, M. R. Galinski, E. O. Voit, Analysis of erythrocyte dynamics in Rhesus macaque monkeys during infection with Plasmodium cynomolgi. Malar. J. 17, 410 (2018).

51. Nonclinical considerations for mitigating nonhuman primate supply constraints arising from the COVID-19 pandemic guidance for industry (United States Food and Drug Administration, 2022); https://digirepo.nlm.nih.gov/master/borndig/9918451285806676/9918451285806676.pdf.

52. A. Roth, S. P. Maher, A. J. Conway, R. Ubalee, V. Chaumeau, C. Andolina, S. A. Kaba, A. Vantaux, M. A. Bakowski, R. Thomson-Luque, S. R. Adapa, N. Singh, S. J. Barnes, C. A. Cooper, M. Rouillier, C. W. McNamara, S. A. Mikolajczak, N. Sather, B. Witkowski, B. Campo, S. H. I. Kappe, D. E. Lanar, F. Nosten, S. Davidson, R. H. Y. Jiang, D. E. Kyle, J. H. Adams, A comprehensive model for assessment of liver stage therapies targeting Plasmodium vivax and Plasmodium falciparum. Nat. Commun. 9, 1837 (2018).

53. R. T. A. Mayadunne, E. Rizzardo, J. Chiefari, Y. K. Chong, G. Moad, S. H. Thang, Living radical polymerization with reversible addition-fragmentation chain transfer (RAFT polymerization) using dithiocarbamates as chain transfer agents. Macromolecules 32, 6977–6980 (1999).

54. Y. A. Ebstie, S. M. Abay, W. T. Tadesse, D. A. Ejigu, Tafenoquine and its potential in the treatment and relapse prevention of Plasmodium vivax malaria: The evidence to date. Drug Des. Devel. Ther. 10, 2387–2399 (2016).

55. G. W. Birrell, K. Van Breda, B. Barber, R. Webster, J. S. McCarthy, G. D. Shanks, M. D. Edstein, Quantification of tafenoquine and 5,6-orthoquinone tafenoquine by UHPLCMS/MS in blood, plasma, and urine, and application to a pharmacokinetic study. Molecules 27, 8186 (2022).

56. C. Vuong, L. H. Xie, B. M. Potter, J. Zhang, P. Zhang, D. Duan, C. K. Nolan, R. J. Sciotti, V. E. Zottig, N. P. Nanayakkara, B. L. Tekwani, L. A. Walker, P. L. Smith, R. M. Paris, L. T. Read, Q. Li, B. S. Pybus, J. C. Sousa, G. A. Reichard, B. Smith, S. R. Marcsisin, Differential cytochrome P450 2D metabolism alters tafenoquine pharmacokinetics. Antimicrob. Agents Chemother. 59, 3864–3869 (2015).

57. P. L. St Jean, Z. Xue, N. Carter, G. C. Koh, S. Duparc, M. Taylor, C. Beaumont, A. Llanos-Cuentas, R. Rueangweerayut, S. Krudsood, J. A. Green, J. P. Rubio, Tafenoquine treatment of Plasmodium vivax malaria: Suggestive evidence that CYP2D6 reduced metabolism is not associated with relapse in the Phase 2b DETECTIVE trial. Malar. J. 15, 97 (2016).

58. O. R. Idowu, J. O. Peggins, T. G. Brewer, C. Kelley, Metabolism of a candidate 8-aminoquinoline antimalarial agent, WR 238605, by rat liver microsomes. Drug Metab. Dispos. 23, 1–17 (1995).

59. K. Y. Lu, E. R. Derbyshire, Tafenoquine: A step toward malaria elimination. Biochemistry 59, 911–920 (2020).

60. J. K. Nair, J. L. Willoughby, A. Chan, K. Charisse, M. R. Alam, Q. Wang, M. Hoekstra, P. Kandasamy, A. V. Kel'in, S. Milstein, N. Taneja, J. O'Shea, S. Shaikh, L. Zhang, R. J. van der Sluis, M. E. Jung, A. Akinc, R. Hutabarat, S. Kuchimanchi, K. Fitzgerald, T. Zimmermann, T. J. van Berkel, M. A. Maier, K. G. Rajeev, M. Manoharan, Multivalent N-acetylgalactosamine-conjugated siRNA localizes in hepatocytes and elicits robust RNAi-mediated gene silencing. J. Am. Chem. Soc. 136, 16958–16961 (2014).

61. A. D. Springer, S. F. Dowdy, GalNAc-siRNA conjugates: Leading the way for delivery of RNAi therapeutics. Nucleic Acid Ther. 28, 109–118 (2018).

62. Q. Li, M. O'Neil, L. Xie, D. Caridha, Q. Zeng, J. Zhang, B. Pybus, M. Hickman, V. Melendez, Assessment of the prophylactic activity and pharmacokinetic profile of oral tafenoquine compared to primaquine for inhibition of liver stage malaria infections. Malar. J. 13, 141 (2014).

63. D. Caridha, M. Hickman, L. Xie, F. Ngundam, E. Milner, A. Schenk, K. Butler, D. Nugent, P. Lee, N. Roncal, S. Leed, E. Hosford, J. Lee, R. J. Sciotti, G. Reichard, C. Black, M. Kreishman-Deitrick, Q. Li, B. Vesely, Updating the modified Thompson test by using whole-body bioluminescence imaging to replace traditional efficacy testing in experimental models of murine malaria. Malar. J. 18, 38 (2019).

64. R. Rochford, C. Ohrt, P. C. Baresel, B. Campo, A. Sampath, A. J. Magill, B. L. Tekwani, L. A. Walker, Humanized mouse model of glucose 6-phosphate dehydrogenase deficiency for in vivo assessment of hemolytic toxicity. Proc. Natl. Acad. Sci. U.S.A. 110, 17486–17491 (2013).

65. N. P. Nanayakkara, B. L. Tekwani, H. M. Herath, R. Sahu, M. Gettayacamin, A. Tungtaeng, Y. van Gessel, P. Baresel, K. S. Wickham, M. S. Bartlett, F. R. Fronczek, V. Melendez, C. Ohrt, G. A. Reichard, J. D. McChesney, R. Rochford, L. A. Walker, Scalable preparation and differential pharmacologic and toxicologic profiles ofprimaquine enantiomers. Antimicrob. Agents Chemother. 58, 4737–4744 (2014).

66. Meeting report of the technical consultation to review the classification of glucose-6-phosphate dehydrogenase (G6PD) (World Health Organization, 2022). Reference Number: WHO/UCN/GMP/MPAG/2022.01. https://who.int/publications/m/item/WHO-UCN-GMP-MPAG-2022.01.

67. S. A. Charman, A. Andreu, H. Barker, S. Blundell, A. Campbell, M. Campbell, G. Chen, F. C. K. Chiu, E. Crighton, K. Katneni, J. Morizzi, R. Patil, T. Pham, E. Ryan, J. Saunders, D. M. Shackleford, K. L. White, L. Almond, M. Dickins, D. A. Smith, J. J. Moehrle, J. N. Burrows, N. Abla, An in vitro toolbox to accelerate anti-malarial drug discovery and development. Malar. J. 19, 1 (2020).

68. K. Kapil, A. M. Jazani, G. Szczepaniak, H. Murata, M. Olszewski, K. Matyjaszewski, Fully oxygen-tolerant visible-light-induced ATRP of acrylates in water: Toward synthesis of protein-polymer hybrids. Macromolecules 56, 2017–2026 (2023).

69. C. Fu, C. Zhang, H. Peng, F. Han, C. Baker, Y. Wu, H. Ta, A. K. Whittaker, Enhanced performance of polymeric 19F MRI contrast agents through incorporation of highly water-soluble monomer MSEA. Macromolecules 51, 5875–5882 (2018).

70. A. J. Debacker, J. Voutila, M. Catley, D. Blakey, N. Habib, Delivery of oligonucleotides to the liver with GalNAc: From research to registered therapeutic drug. Mol. Ther. 28, 1759–1771 (2020).

71. C. Ruwende, S. C. Khoo, R. W. Snow, S. N. Yates, D. Kwiatkowski, S. Gupta, P. Warn, C. E. Allsopp, S. C. Gilbert, N. Peschu, Natural selection of hemi- and heterozygotes for G6PD deficiency in Africa by resistance to severe malaria. Nature 376, 246–249 (1995).

72. E. Beutler, S. Duparc, Glucose-6-phosphate dehydrogenase deficiency and antimalarial drug development. Am. J. Trop. Med. Hyg. 77, 779–789 (2007).

73. Efficacy and safety study of tafenoquine (TQ) co-administered with dihydroartemisininpiperaquine (DHA-PQP) for the radical cure of Plasmodium vivax (P. vivax) malaria. ClinicalTrials.gov Identifier: NCT02802501 (2019); https://ClinicalTrials.gov/show/NCT02802501.

74. J. A. Watson, N. Nekkab, M. White, Tafenoquine for the prevention of Plasmodium vivax malaria relapse. Lancet Microbe 2, e175–e176 (2021).

75. W. Peters, B. L. Robinson, W. K. Milhous, The chemotherapy of rodent malaria. LI. Studies on a new 8-aminoquinoline, WR 238,605. Ann. Trop. Med. Parasitol. 87, 547–552 (1993).

76. A. Bordat, T. Boissenot, N. Ibrahim, M. Ferrere, M. Leveque, L. Potiron, S. Denis, S. Garcia-Argote, O. Carvalho, J. Abadie, C. Cailleau, G. Pieters, N. Tsapis, J. Nicolas, A polymer prodrug strategy to switch from intravenous to subcutaneous cancer therapy for irritant/vesicant drugs. J. Am. Chem. Soc. 144, 18844–18860 (2022).

77. C. W. Olanow, D. G. Standaert, K. Kieburtz, T. X. Viegas, R. Moreadith, Once-weekly subcutaneous delivery of polymer-linked rotigotine (SER-214) provides continuous plasma levels in Parkinson’s disease patients. Mov. Disord. 35, 1055–1061 (2020).

78. N. Marasini, G. Er, C. Fu, C. N. Subasic, J. Ibrahim, M. Skwarczynski, I. Toth, A. K. Whittaker, L. M. Kaminskas, Development of a hyperbranched polymer-based methotrexate nanomedicine for rheumatoid arthritis. Acta Biomater. 142, 298–307 (2022).

79. A. Nan, S. L. Croft, V. Yardley, H. Ghandehari, Targetable water-soluble polymer-drug conjugates for the treatment of visceral leishmaniasis. J. Control. Release 94, 115–127 (2004).

80. P. L. Carl, P. K. Chakravarty, J. A. Katzenellenbogen, A novel connector linkage applicable in prodrug design. J. Med. Chem. 24, 479–480 (1981).

81. G. M. Dubowchik, R. A. Firestone, L. Padilla, D. Willner, S. J. Hofstead, K. Mosure, J. O. Knipe, S. J. Lasch, P. A. Trail, Cathepsin B-labile dipeptide linkers for lysosomal release of doxorubicin from internalizing immunoconjugates: Model studies of enzymatic drug release and antigen-specific in vitro anticancer activity. Bioconjug. Chem. 13, 855–869 (2002).

82. H. D. King, G. M. Dubowchik, H. Mastalerz, D. Willner, S. J. Hofstead, R. A. Firestone, S. J. Lasch, P. A. Trail, Monoclonal antibody conjugates of doxorubicin prepared with branched peptide linkers: Inhibition of aggregation by methoxytriethyleneglycol chains. J. Med. Chem. 45, 4336–4343 (2002).

83. S. O. Doronina, B. E. Toki, M. Y. Torgov, B. A. Mendelsohn, C. G. Cerveny, D. F. Chace, R. L. DeBlanc, R. P. Gearing, T. D. Bovee, C. B. Siegall, J. A. Francisco, A. F. Wahl, D. L. Meyer, P. D. Senter, Development of potent monoclonal antibody auristatin conjugates for cancer therapy. Nat. Biotechnol. 21, 778–784 (2003).

84. X. Hu, H. Jiang, W. Bai, X. Liu, Q. Miao, L. Wang, J. Jin, A. Cui, R. Liu, Z. Li, Synthesis, characterization, and targeted chemotherapy of SCT200-linker-monomethyl auristatin E conjugates. Eur. J. Med. Chem. 216, 113297 (2021).

85. B. S. Pybus, S. R. Marcsisin, X. Jin, G. Deye, J. C. Sousa, Q. Li, D. Caridha, Q. Zeng, G. A. Reichard, C. Ockenhouse, J. Bennett, L. A. Walker, C. Ohrt, V. Melendez, The metabolism of primaquine to its active metabolite is dependent on CYP 2D6. Malar. J. 12, 212 (2013).

86. S. Tachibana, S. A. Sullivan, S. Kawai, S. Nakamura, H. R. Kim, N. Goto, N. Arisue, N. M. Palacpac, H. Honma, M. Yagi, T. Tougan, Y. Katakai, O. Kaneko, T. Mita, K. Kita, Y. Yasutomi, P. L. Sutton, R. Shakhbatyan, T. Horii, T. Yasunaga, J. W. Barnwell, A. A. Escalante, J. M. Carlton, K. Tanabe, Plasmodium cynomolgi genome sequences provide insight into Plasmodium vivax and the monkey malaria clade. Nat. Genet. 44, 1051–1055 (2012).

87. Z. Luo, S. A. Sullivan, J. M. Carlton, The biology of Plasmodium vivax explored through genomics. Ann. N. Y. Acad. Sci. 1342, 53–61 (2015).

88. J. Greaves, D. A. Evans, H. M. Gilles, K. A. Fletcher, D. Bunnag, T. Harinasuta, Plasma kinetics and urinary excretion of primaquine in man. Br. J. Clin. Pharmacol. 10, 399–404 (1980).

89. A. M. Clark, J. K. Baker, J. D. McChesney, Excretion, distribution, and metabolism of primaquine in rats. J. Pharm. Sci. 73, 502–506 (1984).

90. J. K. Baird, 8-Aminoquinoline therapy for latent malaria. Clin. Microbiol. Rev. 32, e00011-19 (2019).

91. SUNLENCA (lenacapavir). Highlights of Prescribing Information (United States Food and Drug Administration, 2022); https://accessdata.fda.gov/drugsatfda_docs/label/2022/215973s000lbl.pdf.

92. VIDAZA (azacitidine for injection). Highlights of Prescribing Information (United States Food and Drug Administration, 2004); https://accessdata.fda.gov/drugsatfda_docs/label/2022/050974s034lbl.pdf.

93. C. Berteau, O. Filipe-Santos, T. Wang, H. E. Rojas, C. Granger, F. Schwarzenbach, Evaluation of the impact of viscosity, injection volume, and injection flow rate on subcutaneous injection tolerance. Med. Dev. Evid. Res. 8, 473–484 (2015).

94. L. F. Ferruccio, C. Murray, K. W. Yee, D. Incekol, R. Lee, E. Paisley, P. Ng, Tolerability of Vidaza (azacitidine) subcutaneous administration using a maximum volume of 3 ml per injection. J. Oncol. Pharm. Pract. 22, 605–610 (2016).

95. Y. Huang, Preclinical and clinical advances of GalNAc-decorated nucleic acid therapeutics. Mol. Ther. Nucleic Acids 6, 116–132 (2017).

96. K. G. Rajeev, J. K. Nair, M. Jayaraman, K. Charisse, N. Taneja, J. O'Shea, J. L. Willoughby, K. Yucius, T. Nguyen, S. Shulga-Morskaya, S. Milstein, A. Liebow, W. Querbes, A. Borodovsky, K. Fitzgerald, M. A. Maier, M. Manoharan, Hepatocytespecific delivery of siRNAs conjugated to novel non-nucleosidic trivalent Nacetylgalactosamine elicits robust gene silencing in vivo. Chembiochem 16, 903–908 (2015).

97. D. K. Ho, C. LeGuyader, S. Srinivasan, D. Roy, V. Vlaskin, T. E. J. Chavas, C. L. Lopez, J. M. Snyder, A. Postma, J. Chiefari, P. S. Stayton, Fully synthetic injectable depots with high drug content and tunable pharmacokinetics for long-acting drug delivery. J. Control. Release 329, 257–269 (2021).

98. Q. Li, L. Gerena, L. Xie, J. Zhang, D. Kyle, W. Milhous, Development and validation of flow cytometric measurement for parasitemia in cultures of P. falciparum vitally stained with YOYO-1. Cytometry A 71A, 297–307 (2007).

99. L. Xie, Q. Li, J. Johnson, J. Zhang, W. Milhous, D. Kyle, Development and validation of flow cytometric measurement for parasitaemia using autofluorescence and YOYO-1 in rodent malaria. Parasitology 134, 1151–1162 (2007).

100. National Research Council (US) Committee for the Update of the Guide for the Care and Use of Laboratory Animals, *Guide for the Care and Use of Laboratory Animals* (National Academies Press, ed. 8, 2011).

101. B. Adulyadef, Animals for Scientific Purposes Act. *Government Gazette*, **132**, Part 18a. Bangkok, Thailand (2015); https://ird.sut.ac.th/ird2020/File/Animal/4.2%20ANIMAL

S%20FOR%20SCIENTIFIC%20PURPOSES%20ACT,%20B.E.%202558%20(A.D.%20 2015).pdf.

102. P. Vanachayangkul, R. Im-Erbsin, A. Tungtaeng, C. Kodchakorn, A. Roth, J. Adams, C. Chaisatit, P. Saingam, R. J. Sciotti, G. A. Reichard, C. K. Nolan, B. S. Pybus, C. C. Black, L. A. Lugo-Roman, M. D. Wegner, P. L. Smith, M. Wojnarski, B. A. Vesely, K. C. Kobylinski, Safety, pharmacokinetics, and activity of high-dose ivermectin and chloroquine against the liver stage of Plasmodium cynomolgi infection in rhesus macaques. Antimicrob. Agents Chemother. 64, e00741-20 (2020).

103. N. Gural, L. Mancio-Silva, A. B. Miller, A. Galstian, V. L. Butty, S. S. Levine, R. Patrapuvich, S. P. Desai, S. A. Mikolajczak, S. H. I. Kappe, H. E. Fleming, S. March, J. Sattabongkot, S. N. Bhatia, In vitro culture, drug sensitivity, and transcriptome of Plasmodium vivax hypnozoites. Cell Host Microbe 23, 395–406.e4 (2018).

104. A. C. Y. Chua, A. Ananthanarayanan, J. J. Y. Ong, J. Y. Wong, A. Yip, N. H. Singh, Y. Qu, L. Dembele, M. McMillian, R. Ubalee, S. Davidson, A. Tungtaeng, R. Imerbsin, K. Gupta, C. Andolina, F. Lee, K. S.-W. Tan, F. Nosten, B. Russell, A. Lange, T. T. Diagana, L. Renia, B. K. S. Yeung, H. Yu, P. Bifani, Hepatic spheroids used as an in vitro model to study malaria relapse. Biomaterials 216, 119221 (2019).

105. D. K. Gupta, L. Dembele, A. Voorberg-van der Wel, G. Roma, A. Yip, V. Chuenchob, N. Kangwanrangsan, T. Ishino, A. M. Vaughan, S. H. Kappe, E. L. Flannery, J. Sattabongkot, S. Mikolajczak, P. Bifani, C. H. Kocken, T. T. Diagana, The Plasmodium liver-specific protein 2 (LISP2) is an early marker of liver stage development. eLife 8, e43362 (2019).

106. K. Hochdorffer, K. A. Ajaj, C. Schafer-Obodozie, F. Kratz, Development of novel bisphosphonate prodrugs of doxorubicin for targeting bone metastases that are cleaved pH dependently or by cathepsin B: Synthesis, cleavage properties, and binding properties to hydroxyapatite as well as bone matrix. J. Med. Chem. 55, 7502–7515 (2012).
